# Contrasting Reproductive Strategies of Two *Nymphaea* Species Affect Existing Natural Genetic Diversity as Assessed by Microsatellite Markers: Implications for Conservation and Wetlands Restoration

**DOI:** 10.3389/fpls.2022.773572

**Published:** 2022-03-09

**Authors:** Seema Parveen, Nutan Singh, Arjun Adit, Suman Kumaria, Rajesh Tandon, Manu Agarwal, Arun Jagannath, Shailendra Goel

**Affiliations:** ^1^Department of Botany, University of Delhi, New Delhi, India; ^2^Department of Botany, North-Eastern Hill University, Shillong, India

**Keywords:** conservation, genetic diversity, microsatellite, population structure, propagules, reproduction

## Abstract

*Nymphaea*, commonly known as water lily, is the largest and most widely distributed genus in the order Nymphaeales. The importance of *Nymphaea* in wetland ecosystems and their increased vulnerability make them a great choice for conservation and management. In this work, we studied genetic diversity in a collection of 90 *N. micrantha* and 92 *N. nouchali* individuals from six different states of India, i.e., Assam, Manipur, Meghalaya, Maharashtra, Goa, and Kerala, using simple sequence repeat (SSR) markers developed by low throughput Illumina sequencing (10X coverage of genome) of *N. micrantha*. *Nymphaea nouchali* is native to India, whereas *N. micrantha* is suggested to be introduced to the country for its aesthetic and cultural values. The study revealed extensive polymorphism in *N. nouchali*, while in *N. micrantha*, no apparent genetic divergence was detected prompting us to investigate the reason(s) by studying the reproductive biology of the two species. The study revealed that *N. micrantha* predominantly reproduces asexually which has impacted the genetic diversity of the species to a great extent. This observation is of immense importance for a successful re-establishment of *Nymphaea* species during restoration programs of wetlands. The information generated on reproductive behaviors and their association with genotypic richness can help in strategizing genetic resource conservation, especially for species with limited distribution. The study has also generated 22,268 non-redundant microsatellite loci, out of which, 143 microsatellites were tested for polymorphism and polymorphic markers were tested for transferability in five other *Nymphaea* species, providing genomic resources for further studies on this important genus.

## Introduction

*Nymphaea*, an aquatic perennial herb commonly known as water lily, is the largest and most widely distributed genus in the order Nymphaeales. The genus is represented by about 45–50 species with a worldwide distribution in the temperate and tropical regions (Borsch et al., 2007). *Nymphaea* is divided into two main groups, Apocarpae and Syncarpiae, which in turn, are divided into five subgenera, *viz.*, *Anecphya, Brachyceras*, and *Hydrocallis, Lotos*, and *Nymphaea*, respectively (Conard, 1905). It is a small group of flowering plants but generates considerable interest in the context of the origin of angiosperms. It belongs to the ANA-grade (Amborellales, Nymphaeales, and Austrobaileyales) of angiosperms, which forms the first three branches of the phylogenetic tree of angiosperms.

*Nymphaea* is popular as an ornamental plant, as well as for food and medicine in many parts of the world. Different parts of the plant, such as rhizome, tuber, leaf, fruit, and seeds, have been used as food in different ways by people in various parts of the world, such as West Africa, Madagascar, Australia, Uganda, Poland, and Central America and Asia (Conard, 1905; Irvine and Trickett, 1953). In many religions prevalent in Asia, such as Hindu and Buddhist religions, water lilies are considered sacred (Ansari and Jeeja, 2009; Yakandawala et al., 2017). In India, it is being favored by plant growers and cultivated in gardens and nurseries throughout the country for its much-admired flower colors (Ansari and Jeeja, 2009). In most freshwater wetlands, *Nymphaea* spp. acts as an important component of plant communities. They provide food and habitat for many aquatic animals and migratory birds, including Siberian cranes and lily-trotters (Jacanas), act to reduce water turbidity and provide in-stream sediment stabilization (Leito et al., 2016; Ranjan and Prakash, 2019; Dalziell et al., 2020). *Nymphaea* species such as *N. mexicana* and *N. violacea* are sensitive to salt stress and are known to occur in freshwater only (Lauer and Ross, 2016; Dalziell et al., 2020). Thus, *Nymphaea* can also act as a significant wetland health indicator (Dalziell et al., 2020). Therefore, the loss of *Nymphaea* plants would be detrimental not only nutritionally and culturally, but also ecologically.

There are 10 species of *Nymphaea* reported from India, of which six (*N. nouchali, N. alba, N. candida, N. pubescens, N. rubra*, and *N. tetragona*) are wild and four (*N. × khurooi, N. caerulea, N. × marliacea*, and *N. micrantha*) are cultivated (Mitra, 1990). The species *N. nouchali, N. rubra*, and *N. pubescens* are distributed throughout the plains of India, whereas others are confined to some areas. Among these, *Nymphaea nouchali*, a wildly growing species, is well known for its medicinal values and *Nymphaea micrantha*, a cultivated species, is well known for its cultural and aesthetic values.

*Nymphaea nouchali* Burm. f. is native to the tropical and subtropical old world to North Australia range, but also found to be distributed in Brazil, Argentina, and South Australia (Plants of the world online; POWO, 2021). It is a well-known medicinal plant, extensively used for the treatment of diabetes, inflammation, liver disorders, urinary disorders, menorrhagia, blennorrhagia, menstruation problem, as an aphrodisiac, and as a bitter tonic (Raja et al., 2010). *Nymphaea micrantha* Guill. and Perr. is native to West Tropical Africa to Chad range (Wiersema, 1988; POWO, 2021), and is known for its cultural and aesthetic values. In India, the *N. nouchali* and *N. micrantha* are the two widely distributed species from the genus *Nymphaea*. *Nymphaea nouchali* is most used for its rhizome and in traditional medicine under Ayurveda and Siddha traditional systems, whereas *N. micrantha* is used for its flowers. In North-East India, the cut flowers of *N. micrantha* are sold in the market and are offered to gods. *Nymphaea nouchali* is native to India (Singh and Jain, 2017), whereas *N. micrantha* is suggested to be introduced as such (Ansari and Jeeja, 2009), although, the source of introduction of *N. micrantha* is not known. These two species were collected to study genetic diversity patterns using microsatellite markers.

The genetic diversity of a species is important for its long-term survival, adaptability, and evolution with changing environmental conditions (Frankham, 2005; Kahilainen et al., 2014). Reduction in allele diversity or heterozygosity can affect the resilience and adaptive potential of a species (Reed and Frankham, 2003). The genus *Nymphaea* is phenotypically diverse and exhibits high levels of inter-specific polymorphism (Heslop-Harrison, 1955). However, studies on molecular genetic diversity and structure within and among populations of *Nymphaea* spp. are limited despite the rich genetic resources worldwide. A limited number of studies have used molecular markers, such as inter simple sequence repeats (ISSR), amplified fragment length polymorphism (AFLP), and random amplified polymorphic DNA (RAPD) for assessing the genetic diversity and resolving relationships among *Nymphaea* species (Woods et al., 2005b; Volkova et al., 2010; Poczai et al., 2011; Dkhar et al., 2013). Genic and inter-genic spacers viz. ITS, chloroplast trnT-trnF regions, matK have also been used for elucidating phylogenetic relationships among different *Nymphaea* species (Woods et al., 2005a; Borsch et al., 2007; Löhne et al., 2008). A study with co-dominant molecular markers with a large number of samples representing diverse geographical areas is missing. The simple sequence repeats (SSRs), because of their high degree of polymorphism and co-dominant nature, are useful in conservation genetics. Their wide genome coverage and efficient transfer to closely related species make them a marker of choice to assess genetic variation and structure among and within populations (Kalia et al., 2011), assigning inbred lines to heterotic groups (Ganapathy et al., 2012; Sajib et al., 2012), and fingerprinting of genotypes (Lörz and Wenzel, 2005).

In this work, we collected 90 *N. micrantha* and 92 *N. nouchali* var. *nouchali* individuals from different regions of India and assessed their genetic diversity and population structure using SSR markers. The specific objectives of the study were: (1) to develop and characterize SSR markers for *Nymphaea* in terms of frequency, genomic distribution, information content, and transferability to related species, and (2) to assess genetic diversity and population structure of the two aforementioned *Nymphaea* species. We discuss the importance of genetic diversity as a resource used to ensure the species success in varied environments. Emphasis is placed on the significance of genetic diversity in the conservation of biodiversity. This is the first report on the genetic diversity and population structure of *Nymphaea* species using genomic SSR markers. Therefore, the genetic information obtained in this study will provide useful resources for various genetic studies in *Nymphaea* and guide strategies to conserve wild species.

## Materials and Methods

### Survey and Collection of Plant Material

Wetland areas of different states of North-East India (Assam, Meghalaya, and Manipur), Western India (Maharashtra, Goa), and Southern India (Kerala) were explored during the years 2017–2020 to survey and collect biological samples of the genus *Nymphaea*. Sixteen populations of *N. micrantha* and 27 populations of *N. nouchali* consisting of 90 and 92 individuals, respectively, were collected from different locations in Assam, Meghalaya, Manipur, Goa, Maharashtra, and Kerala. The species were identified by Prof. Santhosh Nampy and Dr. A. K. Pradeep, University of Calicut, Kerala, and the voucher specimens were deposited at the herbarium of Department of Botany, University of Delhi (DUH14710-DUH14732) and Botanical Survey of India, Eastern Regional Centre, Meghalaya (ASSAM96585-ASSAM96610). The plant material of 3–5 individuals per population was collected, dried in silica, and brought to the laboratory for DNA extraction. To avoid sampling from the same plant and capture more diversity, the samples were collected from the plants growing at a greater distance than 5 m. Individuals collected from a single wetland were assigned to one population. All the accessions studied in the present study are tabulated in Table 1.

**TABLE 1:** Details of plant samples collected and used in this study with their respective geographic locations (latitude and longitude) in six different states of India.

Population ID	Species	Date of Collection	Place of collection	Latitude	Longitude	Sample size	Voucher no.
1S	*Nymphaea nouchali Burm.f. var. nouchali*	02.02.2020	Merces, Goa	15° 29′ 41.41″	73° 50′ 58.54″	4	DUH14710
2S	*Nymphaea nouchali Burm.f. var. nouchali*	03.02.2020	Maina, Goa	15° 26′ 21.96″	73° 52′ 22.56″	3	DUH14711
3S	*Nymphaea nouchali Burm.f. var. nouchali*	03.02.2020	Batim lake, Tiswadi, Goa	15° 27′ 3.34″	73° 53′ 57.44″	1	DUH14712
3S	*Nymphaea nouchali Burm.f. var. nouchali*	03.02.2020	Batim lake, Tiswadi, Goa	15° 26′ 59.77″	73° 53′ 43.9″	2	DUH14713
4S	*Nymphaea nouchali Burm.f. var. nouchali*	03.02.2020	Azossim, Goa	15° 27′ 58.31″	73° 55′ 20.85″	3	DUH14714
5S	*Nymphaea nouchali Burm.f. var. nouchali*	03.02.2020	Naora, Goa	15° 33′ 14.61″	73° 55′ 26.09″	3	DUH14715
6S	*Nymphaea nouchali Burm.f. var. nouchali*	03.02.2020	Mandur, Goa	15° 27′ 2.32″	73° 55′ 22.32″	3	DUH14716
7S	*Nymphaea nouchali Burm.f. var. nouchali*	06.02.2020	Raitollem lake, Curtorim, Goa	15° 16′ 13.43″	74° 1′ 15.09″	3	DUH14717
8S	*Nymphaea nouchali Burm.f. var. nouchali*	06.02.2020	Communidade lake, Guirdolim, Goa	15° 17′ 43.8″	74° 2′ 21.06″	3	DUH14718
9S	*Nymphaea nouchali Burm.f. var. nouchali*	06.02.2020	Kumtalle lake, Goa	15° 17′ 47.62″	74° 2′ 29.3″	3	DUH14719
10S	*Nymphaea nouchali Burm.f. var. nouchali*	06.02.2020	Mulem lake, Goa	15° 13′ 42.56″	74° 1′ 42.01″	3	DUH14720
11S	*Nymphaea nouchali Burm.f. var. nouchali*	05.02.2020	Thakurwadi wetland, Nerurpar, Maharashtra	16° 0′ 32.63″	73° 34′ 7.33″	3	DUH14721
12S	*Nymphaea nouchali Burm.f. var. nouchali*	05.02.2020	Pat lake, Kudal, Maharashtra	15° 58′ 3.02″	73° 34′ 7.33″	4	DUH14722
13S	*Nymphaea nouchali Burm.f. var. nouchali*	05.02.2020	Nerur chavata, Maharashtra	16° 0′ 8.01″	73° 38′ 16.38″	3	DUH14723
K1	*Nymphaea nouchali Burm.f. var. nouchali*	03.03.2020	Ramanattukara, Kerala	11° 12′ 2.2″	75° 52′ 22.12″	3	DUH14724
K2	*Nymphaea nouchali Burm.f. var. nouchali*	03.03.2020	Pantheeramkavu, Kerala	11° 12′ 36.95″	75° 51′ 42.44″	3	DUH14725
K3	*Nymphaea nouchali Burm.f. var. nouchali*	03.03.2020	Koodathumpara, Kozhikode, Kerala	11° 14′ 18.96″	75° 50′ 28.19″	3	DUH14726
K5	*Nymphaea nouchali Burm.f. var. nouchali*	04.02.2020	Malabar botanical garden. Kerala	11° 14′ 21.99″	75° 49′ 40.57″	4	DUH14727
K6	*Nymphaea nouchali Burm.f. var. nouchali*	05.02.2020	Valiyaparappur Thamara kayal, Kerala	10° 52′ 54.9″	75° 59′ 41.1″	3	DUH14728
K7	*Nymphaea nouchali Burm.f. var. nouchali*	05.02.2020	Pallat Kaayal, Kerala	10° 53′ 5.9″	75° 59′ 17.85″	3	DUH14729
K8	*Nymphaea nouchali Burm.f. var. nouchali*	05.02.2020	Triprangode, Kerala	10° 51′ 26.53″	75° 56′ 33.42″	3	DUH14730
A1	*Nymphaea nouchali Burm.f. var. nouchali*	03.06.2018	Krishnai, Assam	26° 1′59.50″N	90°39′53.56″E	4	DUH14731
A2	*Nymphaea nouchali Burm.f. var. nouchali*	03.06.2018	Dudhnoi, Assam	25° 59′9.74″N	90°47′11.73″E	4	DUH14732
A3	*Nymphaea nouchali Burm.f. var. nouchali*	05.06.2017	Batabari, Assam	26°27′27.37″N	90°12′12.12″E	5	ASSAM96586
A4	*Nymphaea nouchali Burm.f. var. nouchali*	05.06.2017	Hajo beel, Assam	26° 15′3.28″N	91°31′35.18″E	4	ASSAM96587
A5	*Nymphaea nouchali Burm.f. var. nouchali*	05.06.2017	Deepor beel, Assam	26° 7′48.00″N	91°39′36.00″E	4	ASSAM96588
A6	*Nymphaea nouchali Burm.f. var. nouchali*	05.06.2017	Bolbolla, Assam	26° 4′53.38″N	90°36′22.35″E	4	ASSAM96585
A7	*Nymphaea nouchali Burm.f. var. nouchali*	03.06.2017	Chaygaon, Assam	26° 2′53.04″N	91°23′12.20″E	4	ASSAM96589
A8	*Nymphaea micrantha Guill* and *Perr.*	06.06.2018	Jorhat (Assam agriculture university), Assam	26°62′18.23″N	94°22′28.27″E	5	ASSAM96607
A9	*Nymphaea micrantha Guill* and *Perr.*	06.06.2018	Jorhat, Assam	26° 79′53.12″N	94°23′10.20″E	5	ASSAM96608
M1	*Nymphaea micrantha Guill* and *Perr.*	25.05.2017	Damrapatpara, Meghalaya	25°56′12.49″N	90°46′55.30″E	5	ASSAM96609
M2	*Nymphaea micrantha Guill* and *Perr.*	25.05.2017	Baghmara, Meghalaya	25°11′36.57″N	90°38′4.67″E	5	ASSAM96610
MN1	*Nymphaea micrantha Guill* and *Perr.*	28.05.2018	Thangmeiband Sinam Leikai, Manipur	24°49′41.41″N	93°56′5.76″E	5	ASSAM96597
MN2	*Nymphaea micrantha Guill* and *Perr.*	28.05.2018	Namthalong, Manipur	24°49′30.24″N	93°56′12.70″E	5	ASSAM96598
MN3	*Nymphaea micrantha Guill* and *Perr.*	28.05.2018	Hiyangthang, Manipur	24°43′32.00″N	93°54′14.45″E	5	ASSAM96599
MN4	*Nymphaea micrantha Guill* and *Perr.*	28.05.2018	Lamphelpat, Manipur	24°49′26.89″N	93°54′39.06″E	5	ASSAM96600
MN5	*Nymphaea micrantha Guill* and *Perr.*	29.05.2018	Bsihnupur, Manipur	24°37′49.30″N	93°45′35.38″E	5	ASSAM96601
MN6	*Nymphaea micrantha Guill* and *Perr.*	29.05.2018	Iskok, Manipur	24°39′1.98″N	93°51′10.10″E	5	ASSAM96590
MN7	*Nymphaea micrantha Guill* and *Perr.*	29.05.2018	Motumyangbi near Iskok, Manipur	24°39′1.78″N	93°51′12.20″E	5	ASSAM96591
MN8	*Nymphaea micrantha Guill* and *Perr.*	29.05.2018	Makha, Manipur	24°42′24.88″N	93°57′16.88″E	5	ASSAM96592
MN9	*Nymphaea micrantha Guill* and *Perr.*	30.05.2018	Near Iskok, Manipur	24°39′5.70″N	93°51′7.26″E	5	ASSAM96593
MN10	*Nymphaea micrantha Guill* and *Perr.*	30.05.2018	Kabrabamleikai, Manipur	24°49′10.95″N	93°56′20.15″E	5	ASSAM96594
MN11	*Nymphaea micrantha Guill* and *Perr.*	30.05.2018	Pangei, Manipur	24°52′9.81″N	93°58′21.14″E	5	ASSAM96595
MN12	*Nymphaea micrantha Guill* and *Perr.*	30.05.2018	Pangei, near Sainik School, Manipur	24°51′35.14″N	93°58′30.03″E	5	ASSAM96596
**Cross-species amplification**							
1	*Nymphaea caerulea*	20.06.2019	Yamuna Biodiversity Park, Delhi	28° 44′ 10.60″	77° 12′ 54.06″	2	-
2	*Nymphaea* x *khooroi*	05.06.2018	North-eastern Hill University, Shillong	25° 36′ 47.90″	91° 53′ 53.37″	2	-
3	*Nymphaea pubescens*	04.02.2020	Malabar Botanical Garden, Kerala	11° 14′ 21.99″	75° 49′ 40.57″	2	-
4	*Nymphaea malabarica*	04.02.2020	Malabar Botanical Garden, Kerala	11° 14′ 21.99″	75° 49′ 40.57″	2	-
5	*Nymphaea rubra*	04.02.2020	Malabar Botanical Garden, Kerala	11° 14′ 21.99″	75° 49′ 40.57″	2	-

*T_a_ (°C), annealing temperature.*

To assess the cross-species transferability of the SSR markers, a few other *Nymphaea* spp. were also collected (Table 1). Three species, *N. Pubescens*, *N. malabárica*, and *N. rubra*, were collected from the germplasm growing in the wetlands of Malabar botanical garden, Kozhikode, Kerala. Two other species viz. *N. × khurooi* (earlier named as *N. alba* var. *rubra*) and *N. caerulea* were collected from North-eastern Hill University (NEHU), Shillong, and Yamuna Biodiversity Park, Delhi, respectively.

### Genomic DNA Extraction

Genomic DNA was extracted from the silica dried leaf tissues brought from the field using CTAB (hexadecyltrimethylammonium bromide) method described by Doyle and Doyle (1987) with a few modifications. The modifications involved an extended incubation of 1 h in CTAB buffer, an extended RNase treatment of 1 h, a phenol-chloroform extraction (phenol:chloroform: isoamyl alcohol; 25:24:1), and final precipitation by adding 1/10 volume of 3 M sodium acetate and an equal volume of chilled ethanol. The quality and quantity of the extracted DNA were estimated by electrophoresis on a 0.8% agarose gel and using a NanoDrop spectrophotometer (NanoDrop, Wilmington, DE, United States). The final concentration of the DNA was adjusted to 50 ng/μl and the DNA was stored at 4°C.

### Genome Size Estimation

The genome size of *N. micrantha* was estimated by flow cytometry. About 1 cm^2^ of the young fresh leaf was used for sample preparation using the CyStain^®^ PI Absolute P kit (Sysmex, Germany) following manufacturer instructions. *Solanum lycopersicum* L. “Stupicke’ polnı’ rane”’ 1C = 0.98 pg (Doležel et al., 1992) was used as an internal standard reference. In brief, the nuclei of the standard and the sample were isolated, stained, and analyzed simultaneously. The nuclear DNA content was determined using the CyFlow^®^ Cube 8 flow cytometer (Sysmex, Germany) for each sample. For both, the standard reference and *N. micrantha* samples were prepared in replicates, and each was run three times with a minimum of 2,500 nuclei count per sample. The fluorescence values were converted to the DNA content using the following formula:


$$\begin{array}{c} nuclearDNAcontent=\frac{meanpositionofsamplepeak}{meanpositionofthepeakofstandard}\\ \;\times DNAcontentofthestandard \end{array}$$

### Genome Sequencing and *de novo* Assembly

The high-quality genomic DNA of *N. micrantha* was used for genome sequencing. The sequencing library was prepared using the NEBNext Ultra DNA library prep kit for Illumina (New England BioLabs, United States) following the manufacturer’s instructions. In brief, the genomic DNA was fragmented by sonication, followed by adaptor ligation (TruSeq Index3 adaptor; GATCGGAAGAGCACACGTCTGAACTCCAGTCACTTAGG CATCTCGTATGCCGTCTTCTGCTTG). The adaptor-ligated fragments were then PCR amplified using NEBnext universal (P3; AATGATACGGCGACCACCGAGATCTACACTCTTTCCCTA CACGACGCTCTTCCGATC*T) and Index3 tagged NEBnext indexed primer (P7; CAAGCAGAAGACGGCATACGAGAT***G CCTAA***GTGACTGGAGTTCAGACGTGTGCTCTTCCGATC) and gel purified. The concentration of the prepared library was quantified by Qubit 4.0 Fluorometer (Thermo Fisher Scientific, United States) and sequenced on Illumina HiSeq 2000 platform in paired-end sequencing mode (Illumina, United States) by Macrogen Inc., (South Korea). The FastQ files containing the raw reads were submitted to the sequence read archive (SRA) at National Centre for Biotechnology Information (NCBI) under the accession number PRJNA750447. The raw data was filtered for high-quality reads [Reads with ≥ 70% HQ bases (*Q* ≥ 20)] using the NGS QC toolkit (Patel and Jain, 2012) at default parameters. High-quality reads obtained were used for *de novo* assembly using Velvet (v1.2.10), with the k-mer length of 55. The assembled sequences were filtered to all contigs greater than or equal to 1,000 bp to remove fragmentation of the assembly.

### Identification of Microsatellites and Functional Annotation

The assembled sequences of the *N. micrantha* genome were mined for perfect microsatellites using MISA (MicroSAtellite identification tool; Thiel et al., 2003). The minimum numbers of repeats used to select the microsatellites were sixteen for mono-, nine for di-, six for tri-, and five for tetra-, penta- and hexanucleotide repeats. For functional annotation, the SSR containing sequences were searched against the NCBI non-redundant (nr) protein sequence database using the BLASTx program with an e-value cutoff 1e^–5^ or better. Further, Gene Ontology (GO) terms were assigned by adopting the terms that had been assigned to the top hit sequences of the database and mapping them to the higher-level categories using the Functional Annotation Tool of DAVID Bioinformatics Resources 6.8 (Huang et al., 2009).

### Primer Design

Sequences harboring SSRs with a minimum repeat length of 20 bases and optimum flanking regions (≥50 nucleotides on both flanks of SSR) were used to design primers. The web-based program, Primer3Plus^1^, was used to design primer pairs from the SSR flanking regions. The parameters for designing primers were—primer length of 18–25 with an optimum of 22 bases; product size range of 100–500 with an optimum of 250 bases; annealing temperature between 5 and 65°C with an optimum value of 60°C and GC content between 40 and 80%, with an optimum of 60%. Other parameters, such as overall self and 3′ self-complementarity, penalty weight, and sequence quality scores, were kept at the default setting. A total of 217 primer pairs were designed and synthesized at Eurofins Genomics India Pvt. Ltd., India (Supplementary Table 1). Additionally, the 43 genic SSR markers were designed from the transcriptomic data (unpublished data, S. Parveen and S. Goel) (Supplementary Table 1).

### Marker Amplification

The 43 genic-SSR, designated as NymTr_1 to NymT_43 and a subset of 100 non-genic SSR markers, designated as Nym_NGS1 to Nym_NGS100 were chosen for experimental validation. These markers were tested for amplification using two *N. micrantha* DNA samples. The PCR reaction was conducted in a total reaction volume of 15 μl containing 1x PCR buffer, 2 mM of MgCl_2_, 0.2 mM of each dNTP, 0.4 mM each of forward and reverse primers, 50 ng of template genomic DNA, and 1.25 U of Taq DNA polymerase (iNtRON Biotechnology, South Korea). The DNA amplification was performed in a SimpliAmp™ thermal cycler (Applied Biosystems, United States) with the following cycling parameters: initial denaturation at 94°C for 3 min followed by 30 cycles at 94°C for 20 s, optimized primer annealing temperature (between 5 and 60°C) for 20 s, DNA extension at 72°C for 30 s, and a final extension at 72°C for 7 min. The amplified products were initially resolved on 2% agarose and visualized under UV-light after ethidium bromide staining.

### Genotyping and Cross-Species Transferability

Primers producing a clear unambiguous band were selected and further analyzed on 6% polyacrylamide gel for all the accessions of *N. micrantha* and *N. nouchali*. Amplicon size was determined by comparing it with a 100 bp DNA ladder (GeneDireX, Inc.). Unambiguously amplified alleles for each SSR locus were scored individually and coded as for their integer size in base pairs (bp). To avoid genotyping errors due to PCR-based amplification failures, the software Micro-Checker 2.2.3 (Van Oosterhout et al., 2004), was used to estimate the errors in allele scoring, presence of null alleles, large allele dropouts, or stuttering during PCR amplification. Null allele frequency (NAF) for each locus was calculated based on the formulas of Chakraborty et al. (1994) and Brookfield (1996). The polymorphic microsatellite markers were further assessed for their cross-species transferability in five *Nymphaea* species viz. *N. pubescence, N. malabarica, N. rubra, N. × khurooi*, and *N. caerulea* (see Supplementary Notes).

### Diversity Analysis of Simple Sequence Repeat Markers

Various statistical genetic diversity estimates such as an average number of alleles (N_a_), overall, an effective number of alleles (N_e_), observed (H_o_) and expected (H_e_) heterozygosity, F_st_ = Nei’s differentiation index (F_st_), and Gene flow (N_m_) were calculated for each locus using GenAlEx 6.5 (Smouse and Peakall, 2012). The polymorphism information content (PI) for each of the primer pairs has been calculated using the package POLYSAT (Clark and Jasieniuk, 2011), with the following formula.


$$PIC=1-\sum_{i=1}^{n}P_{i}^{2}-\sum_{i=1}^{n-1}\sum_{j=i+1}^{n}2P_{i}^{2}P_{j}^{2}$$

Where *n* is the number of alleles, and P_i_ and P_j_ are the frequencies of alleles i and j, respectively (Botstein et al., 1980). Deviations from Hardy–Weinberg equilibrium were estimated for each locus in each population and across all populations based on Fisher’s exact test using default settings in GENEPOP version 4.7.5 (Rousset et al., 2020). The significance of deviations was adjusted using sequential Bonferroni correction (Rice, 1989). Deviation from hardy-weinberg equilibrium (HWE) for each locus in each population was analyzed using a heat plot (Supplementary Figure 1) generated using the PEGAS package in R (Goss et al., 2014). Pairwise linkage disequilibrium (LD) between SSRs was computed based on log-likelihood ratio statistics (G-test) in GENEPOP.

### Genetic Diversity and Population Structure

To estimate within population diversity, the genetic diversity parameters were calculated using GenAlEx 6.5 and poppr R package. Observed mean number of alleles (A_o_), the mean number of effective alleles (N_e_), percentage of polymorphic loci (PPL), number of private alleles (A_p_), and mean observed heterozygosity (H_o_) were calculated using Genalex and the Nei’s unbiased gene diversity/expected heterozygosity (H_e_; Nei, 1978), Shannon–Wiener index of diversity (H; Shannon, 2001), Simpson’s index (lambda; Simpson, 1949), and evenness (E_5_) were calculated using poppr R package. Private alleles were defined as those discovered only in the population considered, discarding those that were found only once as they could reflect genotyping errors.

To evaluate genetic structure in our dataset, we used three different methods: the model-based Bayesian method implemented in the STRUCTURE version 2.3.4 (Pritchard et al., 2010) and maximum likelihood (ML) estimation method implemented in the SNAPCLUST (Beugin et al., 2018); and the model-free DAPC (Discriminant Analysis of Principal Components) (Jombart et al., 2010).

The STRUCTURE uses Markov Chain Monte Carlo (MCMC) approach to estimate every individual’s admixture proportions for a predefined K value (Pritchard et al., 2010). We ran an analysis in the STRUCTURE version 2.3.4 for 150,000 MCMC replications after 50,000 burn-in steps. About 10 replicates each were performed for K values ranging from 1 to 10. The optimum number of populations (K) was estimated using a web-based program, the STRUCTURE HARVESTER (Earl, 2012). The STRUCTURE HARVESTER uses the method outlined by Evanno et al. (2005) and searches for a mode in the ΔK distribution.

The DAPC combines the advantages of principal component analysis (PCA) and discriminant analysis (DA) (Jombart et al., 2010). It first transforms the data using PCA and then performs DA on retained principal components. The number of groups or genetic clusters was defined based on K-means clustering of principal components using find.clusters function in the ADEGENET 2.1.1 package (Jombart, 2008). The optimal K-value was identified by running k-means sequentially for K-value ranging from 1 to 10 and comparing different clustering solutions using BIC (Bayesian Information Criterion). The best-fit K was chosen based on the point at which the elbow in the curve of BIC values as a function of K was observed. The cross-validation function, xvaldapc, was used to determine the minimum number of PCs (Principal Components) to be retained for accurate assignment of every individual to different groups. Finally, the individuals were assigned to clusters based on the posterior membership probabilities of every individual as a measure of the admixture proportion originating in each cluster.

SNAPCLUST allies the advantages of both model-based approaches as used in STRUCTURE and geometric approaches like those used by DAPC and provides a fast maximum-likelihood solution to the specific genetic clustering problem (Beugin et al., 2018). We used Snapclust.choose.k to choose the more adequate number of clusters (K). The most suitable K-value was selected through Bayesian Information Criterion (BIC) by choosing the point at which the BIC value was lowest. After choosing K, we proceeded with the clustering analysis using the function snapclust, implemented in R package ADEGENET.

To further assess the population genetic structure and evaluate the genetic interrelationships among the individuals collected from different locations, unrooted neighbor joining (NJ), and principal coordinate analysis (PCoA) were performed based on pairwise Nei’s genetic distance matrix. The PCoA was generated using dudi. pco function implemented in ade4 R-package (Dray and Dufour, 2007), and the NJ tree was constructed using NJ function from ape R package (Paradis and Schliep, 2019).

### Analysis of Molecular Variance

The analysis of molecular variance (AMOVA) analysis was carried out to test the structure of genetic variation within and among genetic clusters inferred based on population structure analysis.

We performed hierarchical AMOVA analysis using the poppr R package (Kamvar et al., 2014) to quantify the partitioning of variance within the individuals, among individuals within the genetic clusters, and among genetic clusters by using pairwise F_ST_ as the distance measure. Within individual variance was calculated to quantify variation due to heterozygosity within genotype. The F-statistics was calculated to summarize the degree of differentiation at each hierarchical level. The significance of variance components and F-statistics was tested using 9,999 random permutations of data matrices.

### Diversity Analysis Among Genetic Clusters Inferred by Population Structure Analysis

To analyze the dynamics of genetic diversity within each genetic cluster, the measures of genetic diversity were estimated for each cluster obtained during population structure analyses. The genetic diversity indices: observed mean number of alleles (A_o_), the mean number of effective alleles (N_e_), percentage of polymorphic loci (PPL), number of private alleles (A_p_), and mean observed heterozygosity (H_o_) were estimated using GenAlEx 6.5 (Smouse and Peakall, 2012), and Nei’s unbiased gene diversity/expected heterozygosity (He), Shannon–Wiener index of diversity (H), Simpson’s index (lambda), and evenness (E5) were calculated using poppr R package. Weir and Cockerham’s (1984) estimator of pairwise F_st_ between accession groups/clusters were estimated using Hierfstat (Goudet, 2005).

### Isolation by Distance

To determine if the genetic variability is structured in geographic space, we performed an isolation by distance (IBD) test (Mantel, 1967) between geographic populations and within each genetic cluster using procedures implemented in the GenAlEx version 6.5 (Peakall and Smouse, 2006). The Mantel test was performed using the Slatkin’s (Slatkin, 1995) pairwise linearized F_ST_ {F_ST_ [F_ST_/(1 − F_ST_)]} and natural log-transformed geographic distance [ln (1 + geographic distance)] matrix as outlined by Rousset (1997). The statistical significance of the test was conducted by random shuffling (10,000 times) of all individuals among the geographic locations.

The spatial autocorrelation analysis was computed using the multi-locus genetic distance between individuals calculated *via* the method of Peakall et al. (1995) and was further explained in Smouse and Peakall (1999), and the geographic distance calculated as Euclidean distance using the coordinates of the samples’ collection sites (Peakall and Smouse, 2006). The analysis was performed with an even distance class of 6 km and 10,000 permutations. For testing the statistical significance, a two-tailed 95% confidence interval was constructed around the null hypothesis of no genetic structure (*r* = 0). A bootstrap of 10,000 trials was used to estimate a 95% confidence interval around the observed r values for each class.

### Phenology

Besides making a record of pheno-events covering onset/duration of flowering, fruiting, and production of vegetative propagules through regular field visits (2017–2020), the plantlets/propagules were grown in experimental ponds at the Department of Botany, the University of Delhi to monitor the events. The pollinated flowers/fruits (*n* = 20) of both species were collected from the natural populations and dissected to observe the seed set.

### Reproductive Barriers in *Nymphaea micrantha*

As *N. micrantha* exhibited a complete absence of genetic diversity and compromised reproductive output in nature, some of the key aspects of floral biology influencing seed/fruit-set were investigated in the plants growing in experimental ponds at the Department of Botany.

#### Stigmatic and Ovule Receptivity

Flowers were observed from the bud stage onward until the formation of fruits in nature, and the most receptive stage of stigma was ascertained using the peroxidase test (Galen and Plowright, 1987). Besides the time of onset of anthesis, the timing of dehiscence of anther concerning the duration of the stigma receptivity was also noted down. Receptivity of ovules was ascertained by staining them with toluidine blue O’ (*n* = 20; Sengupta and Tandon, 2010).

#### Pollen Biology

Fertility of pollen grains (the ability of the pollen to produce normal male gametes) was determined using 1% acetocarmine (Shivanna and Rangaswamy, 1992), while pollen viability (the ability of the pollen to germinate when conditions are favorable) was determined through fluorochromatic reaction test (Heslop-Harrison and Heslop-Harrison, 1970). For this, fresh pollen grains were extracted from anthers. For each fertility and viability, 20 observations were made using an epifluorescence microscope (Axio plan A.1, Carl Zeiss, Germany) (Supplementary Table 2).

#### Controlled Pollinations

Flowers were either (i) selfed (*n* = 15) or (ii) cross-pollinated (*n* = 15), with fresh pollen on floral stages harboring the most receptive stigma and bagged with a muslin cloth. The pollinated pistils were collected 6 h after pollination (HAP) and fixed in acetic: alcohol (1:3, v/v), cleared with NaOH (1N), and stained with decolorized aniline blue, and visualized under the epifluorescence microscope following Linskens and Esser (1957). A few flowers (*n* = 20) were left untreated (open-pollination) to determine the fruit-set in the plants under cultivation. The pollinated pistils were collected 15 days after pollination (DAP) and dissected to observe a seed set.

## Results

### Genome Sequencing and Discovery of Microsatellites

The genome size estimation of *N. micrantha*, based on the linear relationship of 2C peaks of the sample and the internal standard, indicated an approximate genome size of 1.11 pg per 2C content corresponding to 544 Mbp/1C (Supplementary Figure 2). Illumina paired-end sequencing resulted in 56,645,378 raw reads providing a genome coverage of ∼10 X fold based on the estimated genome size. The raw reads were subjected to quality check to remove sequence artifacts, such as low-quality reads and adapter contamination, using the NGS QC toolkit (Patel and Jain, 2012). A total of 53,511,392 (94.57%) high-quality reads showing a Q value of ≥ 20 were obtained after quality filtration. An assembly of high-quality filtered reads, with Velvet (v1.2.10) at a k-mer value of 55, resulted in 518,464 contigs with an average contig length of 596 bp (Supplementary Table 3). To reduce fragmentation of the assembly, the contigs were further filtered to retain contigs ≥ 1,000 bp resulting in 77,175 contigs with an average contig length of 1,536 bp (Supplementary Figure 3). These contigs were used for the identification of microsatellites. A total of 22,268 non-redundant microsatellite loci were obtained using the Perl script MISA. There were 16,937 sequences containing SSRs of which 4,123 sequences contained more than one SSR. About 2,464 SSRs were present in the compound formation, which were removed from further analysis (see Supplementary Notes and Supplementary Table 4 for details).

### Functional Annotation of Simple Sequence Repeat Containing Sequences

To identify the functional significance of 12,556 non-redundant SSR containing sequences, a BLASTx analysis was performed against the NCBI non-redundant (nr) protein database. A total of 3,809 (30.3%) sequences were found to have similarities to the sequences in the NCBI nr protein database, and 1,623 (12.9%) were found to have at least one functional annotation. The remaining 8,747 (69.67%) did not show significant similarity to the known sequences and were not annotated.

Gene Ontology (GO) enrichment analysis, using DAVID Bioinformatics Resources 6.8 (Huang et al., 2009), classified microsatellite sequences into three categories: cellular components (CC), molecular functions (MFs), and biological process (BP). Supplementary Table 5 provides detailed information regarding annotated SSR sequences. Fourteen hundred and thirty-six contigs were mapped to GO terms with 1,009, 1,172, and 892 assignments distributed under the cellular component, molecular function, and biological process ontology, respectively.

### Genetic Diversity in *Nymphaea micrantha*

The 143 SSR primer pairs were analyzed for polymorphism among 90 *N. micrantha* individuals. Surprisingly, all the markers tested in *N. micrantha* produced the same DNA profile and were monomorphic.

### Genetic Diversity in *Nymphaea nouchali*

#### Genetic Diversity Statistics of Microsatellites

In *N. nouchali*, of 143 SSR markers tested, 50 could not produce clear and scorable bands on PAGE, and 36 were monomorphic and, thus, were excluded from further analysis. The analysis using Micro-Checker software revealed no evidence for stutter error or large allele dropout at any locus for all the populations. The probable presence of null alleles was detected for locus Nym_NGS82 (K1 population) and locus NymTr_41 (K3 population). Accordingly, the adjusted frequencies of amplified alleles for these populations were used for further analysis. Descriptive characterization and genetic diversity analysis among 92 *N. nouchali* individuals, for each of the remaining 57 polymorphic SSR loci, is given in Table 2. A total of 277 alleles were identified with an average of 4.8 alleles per locus. The effective number of alleles (N_e_) ranged between 1.056 (NymTr_13) and 2.339 (NymTr_24), averaging 1.821 alleles per locus. The observed (H_o_) and expected (H_e_) heterozygosity ranged from 0.04 (NymTr_13) to 0.975 (NymTr_21), and 0.03 (NymTr_13) to 0.559 (NymTr_24), respectively. The average observed heterozygosity (H_o_ = 0.578) was higher than the average expected heterozygosity (H_e_ = 0.386). Polymorphic information content (PIC) values varied from 0.042 (NymTr_13) to 0.826 (NymTr_5), with a mean PIC of 0.466. Twenty-six SSR markers were considered as highly informative (PIC > 0.5), twenty-five were moderately informative (0.5 > PIC > 0.3) and six markers (Nym_NGS31, Nym_NGS35, Nym_NGS36, NymTr_42, NymTr_17, and NymTr_13) were considered of little polymorphism information having PIC of < 0.3 values (Table 2). Total gene diversity (H_t_) was highest for the marker NymTr_5 (H_t_ = 0.86), followed by NymTr_31 (H_t_ = 0.751) and Nym_NGS45 (H_t_ = 0.737), and lowest for the marker NymTr_13 (H_t_ = 0.04). Null allele frequencies obtained for all loci were close to zero. No significant linkage disequilibrium was found between the locus pairs based on log-likelihood ratio statistics (G-test). Forty-two out of 57 loci significantly deviated from HWE (*p* < 0.05), of which 35 deviated due to heterozygote excess and 7 loci deviated due to heterozygote deficiency (Table 2). Negative inbreeding coefficient (average F_is_ = −0.456) and deviations from HWE indicate an excess of heterozygosity among *N. nouchali* populations.

**TABLE 2:** Characteristics and genetic diversity indices of 57 polymorphic simple sequence repeat (SSR) markers used for diversity analysis in *Nymphaea nouchali* Burm.f. var. *nouchali*.

Locus	Repeat motif	Forward primer sequence (5′–3′)	Reverse primer sequence (5′–3′)	Size range (bp)	T_a_ (°C)	GenBank Accession No.	N_a_	N_e_	H_o_	H_e_	H_t_	PIC	F_is_	D_HWE_	E_HWE_
Nym_NGS1	(CCA)10	CAGAAGGTTTTCCCTCCTC	TCCCAAAACGCCTATCTCTG	272–278	51	MZ595340	3	1.705	0.644	0.362	0.488	0.425	−0.779	1.0000	0.0000
Nym_NGS7	(TA)8	TCGCATACCCTGAAAGGAAC	CTACCCATCTGTGCCCTACC	126–145	52	MZ595341	10	2.022	0.809	0.480	0.599	0.541	−0.685	1.0000	0.0000
Nym_NGS8	(AAG)10	AGGGTGAGCGTAGCAGAAAG	GCACCACCAAAGGAAACTG	153–165	50	MZ595342	5	1.548	0.459	0.290	0.380	0.340	−0.584	1.0000	0.0003
Nym_NGS11	(GAA)10	CCTAAACCTCCACCCACTTG	GCTTTCAATACCCTCCTCTCC	222–234	52	MZ595343	6	2.156	0.761	0.479	0.656	0.563	−0.589	1.0000	0.0001
Nym_NGS21	(TC)10	CATCCAAACATGCCCATAGA	GACACTGTTCACAATCCAAGAC	274–292	50	MZ595344	7	1.948	0.656	0.435	0.575	0.521	−0.510	0.9949	0.0053
Nym_NGS28	(TC)10	TCATCCAAACATGCCCATAG	CACAATCCAAGACTGCACTT	266–282	50	MZ595345	9	1.935	0.562	0.418	0.632	0.598	−0.343	0.9145	0.0877
Nym_NGS31	(AG)10	GTGCATCTTATCTGGGAGC	GGAAGTGGAAGTGCATAGG	233–237	50	MZ595346	4	1.414	0.309	0.206	0.279	0.241	−0.499	0.9895	0.0312
Nym_NGS33	(T)10	GTTGAGGCACCACCAAACTC	TCTTCAACCGGAGACGGA	279–285	52	MZ595347	4	1.898	0.715	0.435	0.595	0.473	−0.644	1.0000	0.0000
Nym_NGS34	(AG)23	CTCGTGCACATACAAAGTC	AATGCTTGGGAGATGATGG	258–262	50	MZ595348	3	1.919	0.636	0.440	0.556	0.475	−0.445	0.9958	0.0042
Nym_NGS35	(TC)16	CCTGAGAGATCCATTCGAG	TCTGGTGATGGTGATGAG	196–200	51	MZ595349	2	1.132	0.114	0.089	0.108	0.098	−0.278	1.0000	0.5697
Nym_NGS36	(TCT)7	GGCTCAAGCAAGTTCTACC	GGAAATAAGAGGGAGACGG	380–386	51	MZ595350	3	1.461	0.381	0.225	0.335	0.288	−0.696	1.0000	0.0002
Nym_NGS40	(CAC)7	TCTCTCGCTGTCCGTATTCC	TCCTGAACACCCACACTCC	271–280	52	MZ595351	4	1.718	0.616	0.359	0.475	0.422	−0.717	1.0000	0.0000
Nym_NGS45	(GCT)6	GGGCGAAAGTGAAAGAGG	AGTGAGGGCAATGGAAAGG	147–162	50	MZ595352	6	2.098	0.588	0.480	0.737	0.672	−0.223	0.3189	0.6815
Nym_NGS46	(TAA)6	CCCGATACCTGTTACCTG	CCACACTCTTTCTCCTCG	290–302	50	MZ595353	6	1.901	0.492	0.417	0.637	0.513	−0.179	0.2969	0.7038
Nym_NGS51	(GCGACG)5	CCATCTCTCCTCCACCTTTG	ACGCCAAGATTGTCCTCCT	250–350	52	MZ595354	6	2.049	0.430	0.415	0.581	0.509	−0.037	0.0044	0.9958
Nym_NGS53	(CACCGG)5	CCTTCCTTCCTCTTCCAG	CCAGGGGGATCAGGTTATTC	240–252	50	MZ595355	4	2.212	0.914	0.538	0.629	0.540	−0.699	1.0000	0.0000
Nym_NGS54	(TGGGCG)5	CCCACCACAATCCACCTATC	CCCACCACAATCCACCTATC	338–350	51	MZ595356	4	2.144	0.667	0.464	0.567	0.524	−0.437	0.9996	0.0005
Nym_NGS55	(GA)14	AGCATCTACCCGCTCGTAAC	CTCCCTCGTTGCTGCTATTC	290–298	54	MZ595357	4	1.620	0.330	0.293	0.367	0.328	−0.125	0.1750	0.8326
Nym_NGS61	(TTC)5	CTGCGGAGATTGCTCTTC	CTGCGGAGATTGCTCTTC	197–200	52	MZ595358	7	1.738	0.501	0.357	0.657	0.653	−0.401	0.9839	0.0165
Nym_NGS62	(TC)13	GAAACCTGCTTCCGAGTG	GGGTTTCCTCTCAAGTCG	316–328	50	MZ595359	6	1.742	0.488	0.381	0.596	0.552	−0.280	0.7920	0.2132
Nym_NGS63	(AG)12	CAGAACTCACCACAACACC	GGCTTTGAACAACCTGACC	274–290	50	MZ595360	7	1.986	0.601	0.448	0.697	0.659	−0.340	0.9804	0.0197
Nym_NGS64	(TC)15	CCATCTTGCGTCCTCTCTTC	TTAGAGCGGTGGATGGAG	266–274	52	MZ595361	3	1.909	0.781	0.425	0.544	0.434	−0.836	1.0000	0.0000
Nym_NGS65	(T)13	AGCGATGTGTTGGGTTGC	AACTGGTTCAAGCCTCTGC	219–228	52	MZ595362	5	1.989	0.883	0.473	0.579	0.482	−0.866	1.0000	0.0000
Nym_NGS70	(CCT)8	GTTTCCTTGGACCTGTCTCG	AGACCGTCAACATCCTGG	102–199	52	MZ595363	7	1.808	0.485	0.400	0.542	0.497	−0.211	0.5201	0.4839
Nym_NGS71	(AT)7	GCCGAGTCAAACATCTGTCC	CTTCCAAGTCCCAACCTCC	135–141	54	MZ595364	4	1.834	0.512	0.408	0.551	0.446	−0.254	0.7431	0.2600
Nym_NGS72	(GCT)6	GGGCGAAAGTGAAAGAGG	AGTGAGGGCAATGGAAAGG	147–162	54	MZ595365	7	1.982	0.490	0.439	0.694	0.616	−0.116	0.0853	0.9175
Nym_NGS75	(CTT)7	TTCCCACCTTCCTTCTTCC	GAACACCCTTTCCTGTCTTCC	173–182	53	MZ595366	5	2.030	0.840	0.492	0.560	0.483	−0.706	1.0000	0.0000
Nym_NGS76	(TCT)6	GAAGAGGGCAGAGAATGG	TGAAGGCGGGAGTGTAAGAG	269–275	53	MZ595367	6	1.893	0.640	0.426	0.628	0.566	−0.504	0.9679	0.0321
Nym_NGS77	(ACAA)5	CCTGCCAGTTTGCTGTTTC	ACCTCAGCACCCTTCTGTTC	360–375	55	MZ595368	4	2.153	0.901	0.521	0.606	0.559	−0.731	1.0000	0.0000
Nym_NGS82	(TA)14	TCCGTCCTTGCTAACCTG	CCTCAATGTGCTTCCTCACC	300–310	54	MZ595369	6	1.602	0.046	0.308	0.446	0.382	0.850	0.0000	1.0000
Nym_NGS83	(GCAGAT)5	TATTCTGTTCACCCCGTCC	GGCTGGCTGATTTAGTGGAG	251–269	53	MZ595370	4	2.009	0.869	0.499	0.529	0.421	−0.741	1.0000	0.0000
Nym_NGS90	(AAATCC)5	CCGATACGAACACGAACCAC	AGGCATCCACCTCCTCTTCT	290–302	57	MZ595371	4	1.996	0.877	0.480	0.531	0.424	−0.826	1.0000	0.0000
Nym_NGS91	(GAAAGA)5	CGATCCGTCCACAAGTTAGC	CAACAAGGCACGAAGCACT	306–309	56	MZ595372	3	1.956	0.926	0.488	0.602	0.535	−0.899	1.0000	0.0000
Nym_NGS94	(TGGGCG)5	CCCACCACAATCCACCTATC	CCCCTCTTATGCCACAACAC	338–368	50	MZ595373	5	2.264	0.901	0.543	0.639	0.573	−0.660	1.0000	0.0000
NymTr_3	(TA)10	ATCGGGGAAGGAGAAATCAC	TTCAAGAGCAAGCAATCGAC	244–300	56	MZ595374	9	1.700	0.293	0.321	0.453	0.41	0.087	0.0029	0.9969
NymTr_4	(AGA)6	ACATCAGCCTTCCAACTTCC	TTGATGTCCTCGTCCATGTG	203–209	56	MZ595375	2	1.662	0.630	0.353	0.434	0.34	−0.782	1.0000	0.0000
NymTr_5	(AAG)6	TTGAGCAAGGCAGGAGACAC	TGCATCACACGGGTTTAGAAG	185–209	56	MZ595376	12	2.134	0.485	0.416	0.86	0.83	−0.164	0.0925	0.9083
NymTr_9	(GCA)6	TTTCCCTCTGCTCCTGTTTC	TGGAGACGACCTTCCAGTTG	181–190	56	MZ595377	3	2.147	1.000	0.529	0.592	0.5	−0.892	1.0000	0.0000
NymTr_11	(GAG)6	GAAGCCCCAACTTTGAACTG	TCGTCTCCTCCTACCACCTG	177–180	56	MZ595378	2	1.854	0.843	0.438	0.49	0.37	−0.925	1.0000	0.0000
NymTr_12	(GGA)7	ACCTCGGTGAAGTCGCAGT	AAGGGACGAAGATGAAGCTG	226–244	57	MZ595379	5	1.747	0.623	0.384	0.485	0.43	−0.624	1.0000	0.0000
NymTr_13	(ATG)6	TCGATGGGAGAGTTGTGATG	TTCATTCCCCTCAATTCCTC	217–229	57	MZ595380	3	1.056	0.040	0.030	0.04	0.04	−0.339	1.0000	0.5721
NymTr_15	(GCC)7	AGTTTCTGCATCGGAGGTTG	ATCGCGGCTTCTCTTCATAC	183–198	56	MZ595381	6	2.139	0.932	0.514	0.681	0.62	−0.812	1.0000	0.0000
NymTr_16	(CTC)7	TGCCAAGGAAGAGTTCGTG	TCCCTCGATATTCTCAGCAG	192–201	56	MZ595382	3	2.069	0.954	0.502	0.586	0.5	−0.898	1.0000	0.0000
NymTr_17	(CGT)6	GATCAACTCCCGCTATCTCTC	ACGGGCGTGAGGCAGTAG	179–182	56	MZ595383	2	1.117	0.111	0.067	0.105	0.1	−0.655	1.0000	0.1383
NymTr_18	(TCG)6	AGAAGCCCTCTCCTCGACTC	CCATTGACCCCAATTTGTTG	236–242	56	MZ595384	2	1.596	0.556	0.327	0.403	0.32	−0.701	1.0000	0.0000
NymTr_19	(GCT)6	GCATGATTTTCCGGTTCTTG	TGTCGACCTTCGTCAGTGAG	218–227	57	MZ595385	2	1.524	0.478	0.295	0.366	0.3	−0.624	1.0000	0.0001
NymTr_20	(GCT)6	AGCAACCATAATTCGCCTTG	ATCGACCTCATCGTCCAGTC	203–215	57	MZ595386	3	1.486	0.273	0.255	0.578	0.48	−0.072	0.2469	0.7529
NymTr_21	(TGC)6	TCCTGGTGCGATGAGTAGTG	GAAAAGGGATGACGATCTGC	291–297	57	MZ595387	3	2.006	0.975	0.500	0.515	0.39	−0.951	1.0000	0.0000
NymTr_22	(GGT)6	ATGCCTACAGCAGAGGAGGA	TTGCGTAGTGCCATCTGTTC	245–257	56	MZ595388	4	1.975	0.460	0.443	0.652	0.58	−0.040	0.0245	0.9739
NymTr_24	(GTT)6	TGCTCATCATCGTCTTCGTC	CCAAGAAGGAGAAGGACCAG	162–189	57	MZ595389	7	2.339	0.957	0.559	0.717	0.68	−0.712	1.0000	0.0000
NymTr_26	(ACAT)5	ATCCCAACAGACTCCTCCAG	TACCTGCGGAACCCATTAAG	208–224	57	MZ595390	5	1.808	0.569	0.371	0.55	0.51	−0.530	0.9997	0.0002
NymTr_27	(CGTC)5	CTACGGTGGAGGAGGCTATG	CAAAAACCAGACAAGCACCA	283–295	54	MZ595391	2	1.691	0.352	0.364	0.495	0.37	0.035	0.0154	0.9847
NymTr_28	(CTTT)5	TGGCTGTCAAAGAGCATCAG	ACACAGAACCCACCAAATCG	192–208	53	MZ595392	6	1.447	0.117	0.256	0.523	0.48	0.543	0.0000	1.0000
NymTr_30	(GACG)5	CAAAAACCAGACAAGCACCA	GAGGTGATCGCTACCCAAGA	184–200	53	MZ595393	5	1.845	0.494	0.387	0.651	0.59	−0.275	0.7914	0.2151
NymTr_31	(TATG)5	AGCAACAGTTTCACCACCAG	GGTGTATGATAAGGGGGTGTG	219–246	55	MZ595394	7	1.970	0.642	0.427	0.751	0.71	−0.505	0.9909	0.0098
NymTr_41	(GCA)6	AAAATCAGCAAGGGCAACAG	AGCTGGGAGATTTGAGGTTG	148–166	54	MZ595395	5	1.479	0.135	0.228	0.575	0.48	0.408	0.0001	0.9999
NymTr_42	(AGG)6	AGAGGAAGGAGGGAACGAAG	GGAAGAAGAGAAACGCCAGA	158–176	56	MZ595396	3	1.246	0.212	0.132	0.194	0.19	−0.603	1.0000	0.0247

*T_a_ (°C), Annealing temperature; N_a_, No. of Different Alleles; N_e_, No. of Effective Alleles; H_o_, Observed Heterozygosity; H_e_, Expected Heterozygosity; H_t_, Overall gene diversity; PIC, Polymorphic information content; Fis, Inbreeding coefficient; D_HWE_, p-value of deviation from HWE due to heterozygote deficiency; E_HWE_, p-value of deviation from HWE due to heterozygote excess.*

#### Genetic Diversity Analysis Within Populations

Genetic diversity indices obtained for each population of *N. nouchali* are tabulated in Table 3. Average across loci of the observed and effective number of alleles ranged between 1.702 (A2 population) and 2.298 (A1 and A6 populations), and 1.511 (A2 population) to 1.984 (5S population), respectively. The overall mean observed allele number was 2.053 and the effective one was 1.821. The discrepancy between observed and effective allele numbers reflects the uneven distribution of allele frequencies. In total, 277 distinct alleles were identified, of which 32 were private (found only in one population). The population K5 had the maximum number of private alleles (6). The observed heterozygosity (H_o_) was higher than the expected heterozygosity (H_e_) for each population except A6. Ten out of 27 populations showed a significant deviation (*p* < 0.05) from HWE (Table 3 and Supplementary Figure 1). Nei’s unbiased gene diversity ranged between 0.292 (A2) and 0.535 (6S), with an average value of 0.531 across populations. Shannon–Wiener index and Simpson’s Index of genetic diversity suggested the highest diversity in A1 (*H* = 1.609; lambda = 0.8). The genotype abundance/evenness (E_5_) for all the populations except one (K2) indicated that the multi-locus genotypes (MLG) in the populations are equally abundant. More than 50% of the studied loci were polymorphic in all the populations with the highest percentage of polymorphic loci in 6S, K3, and A7 (PPL = 87.72%), and lowest in A2 (PPL = 59.65%).

**TABLE 3:** Genetic diversity statistics of 27 *Nymphaea nouchali* populations.

Population	N	A_o_	N_e_	H_o_	H_e_	H	lambda	E_5_	PPL	A_p_	HWE
1S	4	2.035	1.777	0.592	0.426	1.386	0.750	1.000	82.46%	0	*
2S	3	2.000	1.765	0.509	0.430	1.099	0.667	1.000	75.44%	1	ns
3S	3	1.982	1.818	0.608	0.482	1.099	0.667	1.000	84.21%	1	ns
4S	3	2.035	1.838	0.596	0.489	1.099	0.667	1.000	84.21%	0	ns
5S	3	2.263	1.984	0.567	0.525	1.099	0.667	1.000	85.96%	0	ns
6S	3	2.211	1.969	0.661	0.535	1.099	0.667	1.000	87.72%	0	ns
7S	3	1.965	1.796	0.596	0.441	1.099	0.667	1.000	73.68%	1	ns
8S	3	1.965	1.831	0.602	0.471	1.099	0.667	1.000	78.95%	1	ns
9S	3	2.035	1.779	0.550	0.437	1.099	0.667	1.000	75.44%	1	ns
10S	3	1.947	1.754	0.532	0.448	1.099	0.667	1.000	78.95%	0	ns
11S	3	1.947	1.824	0.637	0.475	1.099	0.667	1.000	80.70%	1	ns
12S	4	2.035	1.808	0.605	0.443	1.386	0.750	1.000	80.70%	1	*
13S	3	1.807	1.703	0.579	0.420	1.099	0.667	1.000	71.93%	0	ns
K1	3	2.088	1.882	0.690	0.498	1.099	0.667	1.000	84.21%	3	ns
K2	3	1.754	1.736	0.702	0.435	0.637	0.444	0.899	71.93%	2	ns
K3	3	2.211	1.979	0.655	0.524	1.099	0.667	1.000	87.72%	3	ns
K5	4	2.123	1.913	0.724	0.487	1.386	0.750	1.000	84.21%	6	*
K6	3	2.018	1.873	0.696	0.494	1.099	0.667	1.000	82.46%	0	ns
K7	3	2.140	1.925	0.661	0.502	1.099	0.667	1.000	85.96%	2	ns
K8	3	1.982	1.789	0.632	0.457	1.099	0.667	1.000	78.95%	1	ns
A1	5	2.298	1.878	0.596	0.449	1.609	0.800	1.000	82.46%	3	*
A2	4	1.702	1.511	0.342	0.292	1.386	0.750	1.000	59.65%	2	*
A3	4	1.825	1.589	0.382	0.317	1.386	0.750	1.000	63.16%	0	*
A4	4	2.316	1.875	0.478	0.461	1.386	0.750	1.000	84.21%	0	*
A5	4	2.088	1.758	0.491	0.430	1.386	0.750	1.000	82.46%	0	*
A6	4	2.298	1.877	0.425	0.432	1.386	0.750	1.000	78.95%	0	*
A7	4	2.351	1.945	0.500	0.489	1.386	0.750	1.000	87.72%	3	*
Mean	NA	2.053	1.821	0.578	0.531	1.196	0.691	0.994	79.79%	NA	NA

*N, sample sizes; A_o_, observed mean no. of alleles; N_e_, no. of effective alleles; H_o_, mean observed heterozygosity; H_e_, Nei’s Unbiased Gene Diversity (Expected Heterozygosity); H, Shannon–Wiener Index of Genotypic Diversity; lambda, Simpson’s Index; E_5_, evenness; PPL, Percentage of Polymorphic Loci; A_p_, Number of Private Alleles; HWE, Deviation from Hardy-Weinberg equilibrium.*

Statistical significance: *p < 0.05; ns, not significant.

Pairwise F_ST_ values ranged between 0.00 (between A4 and A5 populations) and 0.390 (between K2 and A2 populations), showing no genetic differentiation to fairly high genetic differentiation among populations (Table 4).

**TABLE 4:** Multi-locus estimates of pairwise FST between all population pairs of *Nymphaea nouchali*.

	1S	2S	3S	4S	5S	6S	7S	8S	9S	10S	11S	12S	13S	K1	K2	K3	K5	K6	K7	K8	A1	A2	A3	A4	A5	A6
2S	0.045																									
3S	0.107	0.084																								
4S	0.150	0.103	0.039																							
5S	0.105	0.091	0.067	0.024																						
6S	0.173	0.175	0.119	0.071	0.000																					
7S	0.243*	0.218*	0.212	0.194	0.107	0.122																				
8S	0.158	0.140	0.132	0.106	0.096	0.119	0.101																			
9S	0.208*	0.203*	0.162	0.123	0.073	0.104	0.157	0.133																		
10S	0.252*	0.222*	0.197	0.136	0.074	0.134	0.213	0.209	0.107																	
11S	0.198	0.161	0.103	0.150	0.105	0.138	0.184	0.183	0.175	0.128																
12S	0.201*	0.201*	0.171	0.183*	0.112	0.098	0.143	0.182	0.149	0.145	0.088															
13S	0.241*	0.219*	0.177	0.193	0.126	0.155	0.161	0.205	0.188	0.202	0.120	0.065														
K1	0.173	0.182	0.148	0.180	0.149	0.173	0.284*	0.229	0.246*	0.252*	0.194	0.224*	0.253													
K2	0.215	0.251*	0.254*	0.247*	0.184	0.247	0.337*	0.304*	0.301*	0.306*	0.263	0.289*	0.335*	0.169												
K3	0.152	0.178	0.145	0.156	0.099	0.136	0.237*	0.210*	0.187	0.232*	0.176	0.167	0.197	0.045	0.140											
K5	0.225*	0.266*	0.227*	0.230*	0.157	0.167	0.264*	0.271*	0.248*	0.264*	0.257*	0.227*	0.282*	0.202	0.226	0.109										
K6	0.205	0.234*	0.209	0.207	0.160	0.180	0.309*	0.242*	0.240*	0.277*	0.247	0.239*	0.279*	0.165	0.253	0.139	0.199									
K7	0.180	0.188	0.191	0.183	0.133	0.139	0.272*	0.222*	0.221*	0.237*	0.229*	0.207*	0.247*	0.141	0.189	0.111	0.190	0.057								
K8	0.194	0.241*	0.228*	0.240*	0.172	0.196	0.306*	0.265*	0.254*	0.276*	0.234	0.245*	0.289*	0.148	0.199	0.093	0.217*	0.137	0.085							
A1	0.177*	0.206*	0.208*	0.202*	0.146*	0.185*	0.265*	0.246*	0.226*	0.268*	0.230*	0.226*	0.246*	0.178*	0.202*	0.128	0.216*	0.157	0.150	0.142						
A2	0.328*	0.364*	0.356*	0.349*	0.224*	0.288*	0.359*	0.358*	0.311*	0.324*	0.344*	0.317*	0.367*	0.333*	0.390*	0.288*	0.338*	0.342*	0.335*	0.315*	0.217*					
A3	0.294*	0.327*	0.295*	0.277*	0.197*	0.251*	0.305*	0.315*	0.285*	0.313*	0.304*	0.273*	0.321*	0.300*	0.367*	0.254*	0.304*	0.349*	0.344*	0.318*	0.249*	0.080				
A4	0.194*	0.214*	0.185*	0.135	0.070	0.103	0.160*	0.135	0.101	0.166*	0.184*	0.182*	0.211*	0.222*	0.271*	0.177*	0.225*	0.227*	0.229*	0.214*	0.167*	0.132*	0.041			
A5	0.225*	0.249*	0.215*	0.215*	0.143*	0.170*	0.210*	0.192*	0.148*	0.189*	0.224*	0.218*	0.266*	0.249*	0.315*	0.233*	0.276*	0.270*	0.270*	0.254*	0.186*	0.157*	0.119	0.000		
A6	0.213*	0.204*	0.169*	0.190*	0.129*	0.176*	0.210*	0.212*	0.157*	0.184*	0.122	0.148*	0.174*	0.218*	0.285*	0.179*	0.267*	0.249*	0.251*	0.215*	0.183*	0.170*	0.089	0.013	0.045	
A7	0.127*	0.159*	0.174*	0.189*	0.130*	0.170*	0.252*	0.201*	0.217*	0.225*	0.206*	0.214*	0.238*	0.167*	0.234*	0.141*	0.228*	0.159*	0.132	0.105	0.111*	0.195*	0.185*	0.113*	0.146*	0.114*

#### Analysis of Population Structure

**FIGURE 1:**
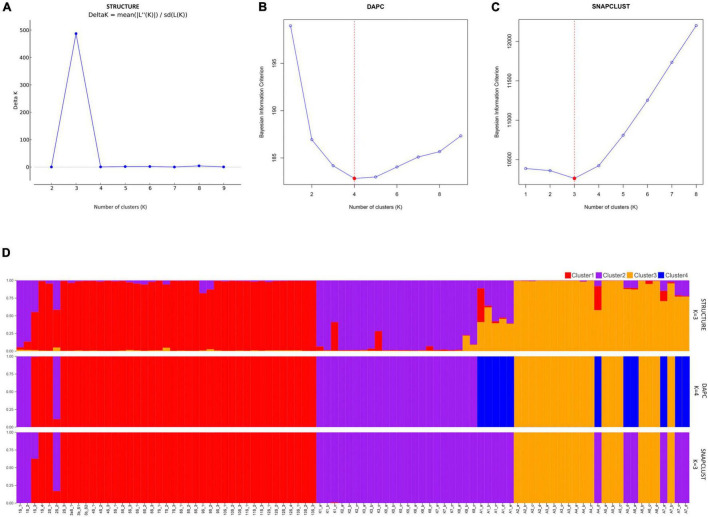
Structure clustering results of population structure analysis. Each inferred cluster is marked with a different color and everyone in the cluster is represented by a vertical bar. Estimation of populations [(K; **(A–C)**] and distribution of individuals to different clusters at *K* = 3 and *K* = 4 **(D)** using STRUCTURE, Discriminant Analysis of Principal Components (DAPC), and SNAPCLUST.

An analysis using both STRUCTURE and SNAPCLUST recovered three clusters with some degree of correlation between genetic clusters and geographical origin (Figures 1A,C). Although the assignment of some individuals to clusters differed between the two methods, in general, there is a high degree of correspondence between the groups defined by SNAPCLUST and those recovered by STRUCTURE (Figure 1D). Accessions from Goa and Maharashtra, which are less geographically distant, were clustered together (cluster 1). All the accessions from Kerala were assigned to cluster 2. Some accessions of Assam were recovered as admixtures (STRUCTURE) and/or groups with the accessions of Kerala (SNAPCLUST) suggesting movement of genetic material between the two geographical locations. The STRUCTURE failed to classify more individuals (11 were classified as admixed based on the threshold defined) as compared to SNAPCLUST which classified individuals into different clusters. The SNAPCLUST placed all individuals into a single group, except one (1S_3) that could not be assigned to a single group with a group membership score of over 0.75.

The DAPC recovered four genetic clusters (Figure 1B), two of which roughly corresponded to the two major groups identified using both STRUCTURE and SNAPCLUST. Unlike STRUCTURE and SNAPCLUST, DAPC classified accessions collected from Assam into two different genetic clusters (cluster 3 and cluster 4; Figure 1D). No individual was classified as admixed based on the threshold defined for this method. All the individuals from Goa and Maharashtra formed a single group except 1S_1, 1S_2, and 2S_2 that were grouped with Kerala.

**FIGURE 2:**
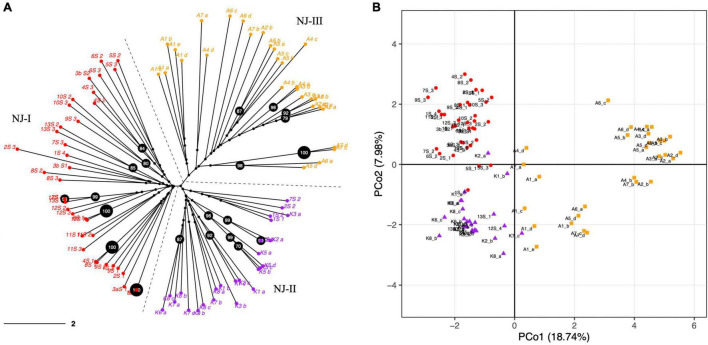
**(A)** Dendrogram constructed with a neighbor-joining (NJ) clustering algorithm elucidating the genetic relationships among 92 *N. nouchali* individuals using 57 polymorphic SSR markers. The NJ clusters (NJ-I, NJ-II and NJ-III) are demarcated through dashed lines and different tip colors. Percentage bootstrap values were generated from 1,000 replicates. Bootstrap values ≥ 70% are depicted in black circles. Scale bar represents branch length. **(B)** Principal Coordinate Analysis (PCoA) exhibiting the distribution of *N. nouchali* accessions on two main axes. Primary axes 1 and 2 captured 18.74% and 7.98% of the total variance, respectively. Different colors represent accessions from different NJ clusters.

The PCoA and NJ were conducted to further assess the population’s genetic structure. Both NJ and PCoA showed a grouping of accessions according to their geographical distances (Figure 2). The NJ analysis (Figure 2A) showed that the accessions from 27 natural populations could be divided into three groups, which was consistent with the pattern from the population structure analysis using STRUCTURE and SNAPCLUST software. Accessions from Maharashtra and Goa were clustered together. The accessions from Kerala were less distant to the accessions from Goa and Maharashtra than those from Assam. In addition, the results of PCoA analysis further verified the NJ and the population structure analysis results (Figure 2B). In summary, the populations with less geographic distance are grouped. The first two principal coordinates explained 18.74% and 7.98% individually and explained 26.72% of the total variation (Figure 2B).

#### Analysis of Molecular Variance

Considering the clustering of accessions into genetic groups, between *K* = 3 and *K* = 4, the AMOVA accounted for more variance for *K* = 4 than *K* = 3 (Table 5). The AMOVA results indicated that most of the total genetic variance (>90%) was attributed to the heterozygosity within individuals (Table 5). Variance attributed to individuals within the genetic cluster was zero with no statistical support and negative F_IS_ value (F_IS_ = −0.161, *p* > 0.05). Significant genetic differentiation (*p* < 0.001) was detected among genetic clusters for both *K* = 3 and *K* = 4 genetic clusters (Table 5).

**TABLE 5:** Analysis of molecular variance (AMOVA) showing variance percentage explained by four (*K* = 4) and three (*K* = 3) genetic clusters inferred by different structure methods.

Source of variation	df	SS	MS	Variance	Percentage of variation	F-statistics	*p-*value
***K* = 4**							
Between genetic clusters	3	237.589	79.196	1.558	9%	F_ST_ = 0.100	<0.001
Between individuals within genetic cluster	88	1035.694	11.769	0.000	0%	F_IS_ = −0.161	>0.05
Within individuals	92	1498.000	16.283	16.283	91%	F_IT_ = −0.045	>0.05
Total	183	2771.283		17.840	100%		
***K* = 3**							
Between genetic clusters	2	205.740	102.870	1.499	8%	F_ST_ = 0.096	<0.001
Between individuals within genetic cluster	89	1067.543	11.995	0.000	0%	F_IS_ = −0.152	>0.05
Within individuals	92	1498.000	16.283	16.283	92%	F_IT_ = −0.041	>0.05
Total	183	2771.283		17.781	100%		

*df, degrees of freedom; SS, sum of squares; MS, mean squares. Significance of tests (p-value) was performed with 9,999 permutations.*

#### Diversity Analysis Among Genetic Clusters

Genetic diversity statistics obtained for each genetic cluster are tabulated in Table 6. The observed heterozygosity (H_o_) and Nei’s unbiased gene diversity (H_e_) were highest in cluster II (H_o_ = 0.667; H_e_ = 0.520) and lowest in cluster IV (H_o_ = 0.463; H_e_ = 0.383). Simpson’s index (H) and Shannon–Wiener index were highest in cluster I (lambda = 0.973; *H* = 3.611) and lowest in cluster III (lambda = 0.909; *H* = 2.398). The genotype abundance/evenness (E_5_) in each cluster was either 1 or close to 1 which indicates the equal abundance of all multi-locus genotypes (MLGs) in these genetic clusters. More than 90% of the loci studied were polymorphic in all four clusters with the highest percentage of polymorphic loci in cluster II (98.25%). All the accession groups amplified private alleles (A_p_) varying between 6 and 30 with the highest number of A_p_ detected in cluster II.

**TABLE 6:** Indices of genetic diversity and pairwise Fst among four genetic clusters.

Accession groups	Indices of genetic diversity									
	N	A_o_	N_e_	H_o_	H_e_	H	Lambda	E_5_	PPL	A_p_
Cluster I	37	3.386	2.204	0.588	0.504	3.611	0.973	1.000	96.49%	16
Cluster II	26	3.491	2.282	0.667	0.520	3.205	0.958	0.978	98.25%	30
Cluster III	11	2.791	1.822	0.466	0.516	2.398	0.909	1.000	94.74%	6
Cluster IV	18	3.298	2.069	0.463	0.383	2.890	0.944	1.000	92.98%	14
Total	92	3.224	2.094	0.546	0.531	4.507	0.989	0.994	95.61%	50

**Pairwise F_ST_ values between genetic clusters**				

	**Cluster I**	**Cluster II**	**Cluster III**	**Cluster IV**

Cluster I	NA			
Cluster II	*0.068	NA		
Cluster III	*0.136	*0.115	NA	
Cluster IV	*0.102	*0.131	*0.053	NA

N, sample sizes; A_o_, observed mean no. of alleles; N_e_, No. of effective alleles; H_o_, mean observed heterozygosity; H_e_, Nei’s unbiased gene diversity (expected heterozygosity); H, Shannon–Wiener Index of Genotypic Diversity; lambda, Simpson’s Index; E_5_, evenness; PPL, Percentage of Polymorphic Loci; A_p_, Number of Private Alleles. *represents statistical significance of F_ST_ values (p < 0.01).

The pairwise F_st_ values between clusters ranged from 0.053 to 0.136, and all the cluster pairs were significantly differentiated *p*-value < 0.01 for each pair (Table 6) from each other. Accessions of cluster III and cluster IV were least differentiated (F_ST_ = 0.053), followed by accessions from cluster I and cluster II (F_ST_ = 0.068). Cluster I and II accessions were more distinct from cluster III and IV accessions than they were from each other, and vice-versa.

#### Spatial Genetic Analysis

**FIGURE 3:**
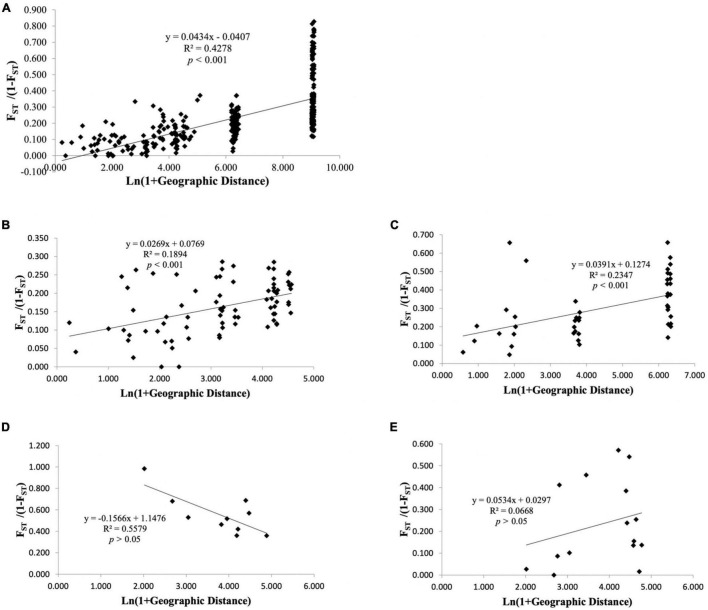
Relationship between Slatkin’s linearized pairwise F_ST_ and geographical distance (Isolation-by-distance based on Mantel test) among populations of *Nymphaea* nouchali **(A)** and genetic clusters inferred using DAPC: cluster I **(B)**, cluster II **(C)**, cluster III **(D)**, and cluster IV **(E)**. Significance of the test was tested based on 10,000 random permutations of the data.

For all geographical locations, as expected under the isolation-by-distance (IBD) model, significant positive correlation was observed between pairwise F_ST_ and geographical distance based on Mantel permutation test (Figure 3A; *r* = 0.654, *p* < 0.001, permutations = 1,000). The multivariate spatial autocorrelation analysis displayed positive and significant r values up to 12 km (Supplementary Figure 4) illustrating that those individuals, which are less than 12 km apart, had non-random genetic similarities.

Among the four genetic clusters inferred with population structure analysis, isolation by distance pattern could be observed within cluster I (Figure 3B; *r*^2^ = 0.187, *p* < 0.001) and cluster II (Figure 3C; *r*^2^ = 0.259, *p* < 0.01), which mostly included the localities from Goa-Maharashtra and Kerala respectively. The multivariate spatial autocorrelation analysis within the genetic groups displayed significant r values for cluster I and suggested a non-random genetic similarity between individuals who are less than 32 km apart. For clusters II, III, and IV, the r values fell within the confidence interval of the null hypothesis (Supplementary Figure 4) suggesting that the individuals within these clusters had random genetic similarities.

### Phenology

**FIGURE 4:**
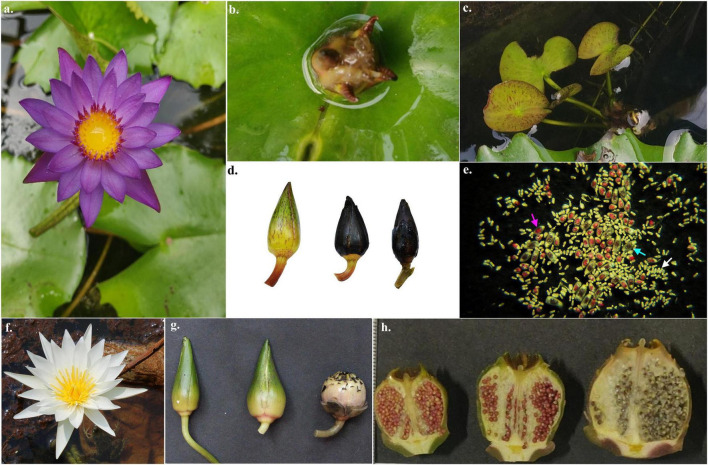
Images showing **(a)** first day flower of *Nymphaea micrantha*, **(b)** bulbil arising from the petiolar node of *N. micrantha*, **(c)** plantlet produced from the bulbil in *N. micrantha*, **(d)** pollinated flower pods of *N. micrantha* after 5, 15, and 20 days of pollination (DAP), **(e)** content of a locule extracted from 15 DAP pod of *Nymphaea*, showing mature seed (blue arrow), aborted seeds (pink arrow) and unfertilized ovules (white arrow), **(f)** flower of *N. nouchali*, and **(g,h)** fruits of *N. nouchali*, collected 5, 15, and 20 DAP.

The flowering and fruiting in *N. nouchali* were observed from March to October; while the *N. micrantha* flowers throughout the year, with peak flowering residing between April and September. Vegetative propagules were observed only in the case of *N. micrantha* arising from the petiolar node (Figures 4b,c). They appeared throughout the year, with copious production during the winters (November–January). Fruits of *N. nouchali* matured within 25 DAP (Figure 4g,h) and dehisced underwater, with multiple longitudinal splits in the capsule, producing an enormous number of seeds. On the contrary, fruit formation in the naturally growing plants of *N. micrantha* was not apparent (Figure 4).

### Reproductive Barrier in *Nymphaea micrantha*

The flowers exhibited protogyny, as the stigma became receptive on the day of anthesis, while anthers dehisced a day later. There was a gradual decline in the receptivity after the first day, indicating overlapping dichogamy in the species. Pollen tubes reached the ovules 6 HAP (Hours after pollination). However, only 4.33 ± 1.91% of the ovules showed receptivity. The fertility of pollen grains was significantly greater (87.89 ± 5.18%) than the viability (18.02 ± 4.51%; *t* = 44.36, df = 38, *p* < 0.001). Controlled pollinations (both selfed and outcrossed) resulted in the initiation of fruits formation but failed to mature into fruits. The pollinated flowers turned brown and started decomposing after 12–15 days of the anthesis phase (Figure 4d). Such abortive fruits were dissected to ascertain seed formation, showed a 2.8 ± 0.84 % seed set (Figure 4e). No difference in seed set was observed between selfed and cross-pollinated flowers. The results of seed set in the naturally growing populations were also comparable with manual pollination treatment.

## Discussion

Aquatic plant species, inhabiting shallow lakes and river ecosystems, are most vulnerable to various anthropogenic disturbances (Guo et al., 2019). The various environmental changes, such as water pollution, habitat destruction, eutrophication, or changed hydrological regime, have led to the global declination and extinction of many aquatic species during the past 50 years (Sand-Jensen et al., 2000; Zhang et al., 2017; Orsenigo, 2018), making them priority targets for conservation. *Nymphaea* species are the important keystone species within the freshwater wetlands. They provide food and habitat for many herbaceous animals, act to reduce water turbidity and provide in-stream water stabilization (Leito et al., 2016; Ranjan and Prakash, 2019). Fruits and seeds of *N. nouchali* are eaten by many birds including Red-crested Pochard, Eurasian Wigeon, Spot-billed Duck, and others (Jha, 2013). In India, the wetland ecosystems are under tremendous stress due to rapid increases in industrialization, urbanization, and agricultural intensification (Bassi et al., 2014). Supplementary Figure 5 shows how much human activities have led to the rapid destruction of water bodies and induced rapid decline of many aquatic plants including *Nymphaea* species.

An assessment of genetic diversity within a species plays an important role in the efficient management and conservation of genetic resources. This study is the first to utilize SSR markers in molecular characterization of two Indian representative species, *N. micrantha* and *N. nouchali*, in their natural habitat from different environmental regions. The investigation used 43 genic SSRs and 100 non-genic SSRs to analyze a collection of 90 *N. micrantha* and 92 *N. nouchali* individuals from six different states of India, i.e., Assam, Manipur, Meghalaya, Maharashtra, Goa, and Kerala. The *N. micrantha*, being an introduced species to India, is expected to possess low genetic diversity, although, to our surprise, the SSR markers revealed no apparent genetic divergence in the studied populations. On the contrary, *N. nouchali* possessed a high level of polymorphism among and within populations.

### Genotypic Richness and Genetic Relationships Among and Within Populations of *N. nouchali* Revealed by Simple Sequence Repeats

#### High Genetic Diversity Within 27 Geographically Distinct Populations of *N. nouchali*

A high level of genetic diversity was observed in *N. nouchali*. The 57 polymorphic SSR markers detected a total of 277 alleles, with an average of 4.8 alleles per locus. The alleles observed for each locus were not equally frequent as the number of alleles observed (A_o_) were greater than an effective number of alleles (A_e_). The negative inbreeding coefficient (mean F_is_ = −0.456) values indicate an excess of heterozygosity in the studied populations of *N. nouchali.* The average total gene diversity (H_t_ = 0.531) among all polymorphic SSR markers was higher than polymorphic information content (PIC = 0.466) suggesting that it is less likely that the *N. nouchali* individuals have identical heterozygote genotypes (Table 2).

Various studies (Gupta, 1978; Hossain et al., 2007) have reported polyploidy in the genus *Nymphaea*. The reported chromosome count in *N. nouchali* varies between 2n = 28 and 2n = 84 (Hossain et al., 2007). Polyploid populations are expected to exhibit high levels of heterozygosity (Muller, 1914; Haldane, 1930; Soltis and Soltis, 2000). In our study, we observed high levels of heterozygosity, but it is not completely fixed. Moreover, the SSR data did not reveal any sign of polyploidy as a maximum of two alleles were present for each locus within an individual.

The average gene diversity (H_e_) within populations ranged between 0.292 and 0.535 (Table 3). The preservation of this high level of genetic diversity in *N. nouchali* may be due to prevailing cross-pollination in the species (Stebbins, 1957). Since *Nymphaea* flowers are protogynous, many *Nymphaea* spp. utilize xenogamy or geitonogamy as their reproductive strategy (Wiersema, 1988). In 1988, Wiersema reported that *N. nouchali* predominantly utilizes xenogamy (i.e., the union of genetically unrelated organisms within a species), which might also explain the observed high within-population gene diversity in the species. Several studies on other aquatic plants, however, have reported low genetic variation (Tian et al., 2008; Lambertini et al., 2010; Hu et al., 2012) based on various dominant molecular markers. Han et al. (2007) had reported high genetic diversity in populations of Nelumbo nucifera using ISSR markers. 42 of 57 polymorphic SSR markers was detected in HWE with *p* ≤ 0.05 (Supplementary Figure 1 and Table 2) in ten populations (Supplementary Figure 1 and Table 3), which included one population each from Goa (1S), Maharashtra (12S), and Kerala (K5), and all the seven populations of Assam. Simpson’s index and Shannon–Wiener index of gene diversity also revealed high levels of genotypic richness within the studied populations (Table 3). The excess of heterozygosity observed in *N. nouchali* populations may be attributed to: (1) the low effective size of populations studied, (2) the outcrossing breeding system, and (3) selection favoring heterozygotes.

#### Population Structure and Genetic Clustering

To estimate genetic populations within a group of *N. nouchali* accessions, we used three different approaches (STRUCTURE, SNAPCLUST, and DAPC). These approaches recovered three or four different clusters with a slightly different partitioning of genotypes into genetic clusters. All the three approaches recovered a high degree of correspondence between genetic clusters and geographical origin, thus, indicating that the individuals with less geographical distances were more genetically similar than those from a greater geographical distance.

Furthermore, the NJ clustering algorithm clustered individuals into three major clusters with correspondence to their geographic origin (Figure 2). Individuals with less geographical distance were clustered together implying that geographical isolation restricted gene exchange among populations (Geraldes et al., 2014; Chen et al., 2020). Estimation of pairwise F_ST_ between each population pair revealed that populations collected from Assam were more differentiated from the populations collected from Goa, Maharashtra, and Kerala (Table 4), which again indicates that populations with less geographic distance were more genetically similar.

The Isolation-by-distance (IBD) pattern also suggested restricted dispersal and regional equilibrium between gene flow and genetic drift in controlling the distribution of genetic variation (Hutchison and Templeton, 1999). The detectable similarity among individuals at a distance < 12 km within some regions, again, indicates that gene flow is effective only over very short distances (Figure 3 and Supplementary Figure 4).

Hierarchical AMOVA analysis was used to quantify the partitioning of variance within the individuals, among individuals within the genetic clusters, and among genetic clusters for both *K* = 4 and *K* = 3. The highest variance was present within geographical among individuals, followed by among genetic clusters and zero variance was detected among individuals within the genetic cluster (Table 5). High within individual variance suggests that most of the observed variation in *N. nouchali* was attributed to the heterozygosity within individuals which might have resulted due to prevalent cross-pollination or xenogamy as a mode of reproductive strategy.

#### Genetic Diversity Measures Within Each Genetic Cluster

**FIGURE 5:**
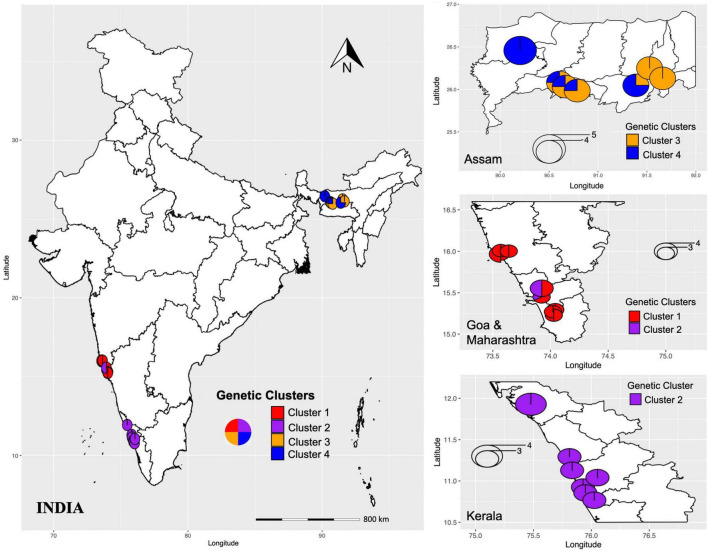
Geographical distribution of genetic clusters (I-IV) inferred by DAPC. The colors represent different genetic clusters and pie charts represent the percentage of individuals assigned to each genetic cluster.

The analysis of genetic diversity within each genetic cluster inferred from DAPC based on three indices (Nei’s unbiased genetic diversity, Shannon–Weiner index of genetic diversity, and Simpson’s index) indicated that the populations collected from southern Indian states, Goa, Maharashtra, and Kerala (represented in clusters I and II; Figure 5) were more genotypically rich as compared to Assam (represented in clusters III and IV; Figure 5). Further, the observed mean number of alleles, percentage of polymorphic loci, and private alleles were also high in clusters I and II as compared to clusters III and IV. These observations suggest (1) predominant outcrossing and a great pre-existing genetic variability (Stebbins, 1957), and (2) low anthropogenic influence in Goa, Kerala, and Maharashtra as compared to Assam. The degree of differentiation can be explained by many factors such as habitat destruction, breeding system type, geographic isolation, and gene flow (Shen et al., 2017). Pairwise F_ST_ between each cluster pair, significantly differentiated (*p* < 0.01) the four clusters. The pairwise F_ST_ values between the clusters showed greater genetic differentiation with increased geographic distance between the two clusters. Clusters I and II individuals were more genetically differentiated from clusters III and IV individuals than from each other. The cluster III and cluster IV individuals, which includes individuals from Assam, were least differentiated (F_ST_ = 0.053), followed by cluster I and cluster II (F_ST_ = 0.068), which included individuals from Goa and Maharashtra. The regional population genetic structure and weak or lack of IBD within clusters (Figure 3) indicated higher gene flow between populations that are less geographically distant.

### Cross-Species Transferability of Polymorphic Simple Sequence Repeat Markers

The 57 polymorphic SSR markers produced for *N. nouchali* were also assessed for their cross-species transferability in five other *Nymphaea* spp. viz. *N. malabarica, N. caerulea, N. rubra, N. pubescens*, and *N. × khooroi* (see Supplementary Table 6). The markers with a high transferability rate would be of importance and can be efficiently utilized in conducting basic genetic studies in *Nymphaea*.

### Clonal Reproduction and Lack of Genotypic Diversity in *N. micrantha* in the Studied Regions

*Nymphaea micrantha* is non-native to India and, thus, is expected to possess low genetic variation. Surprisingly, however, no genetic variation was found within or between populations investigated as a single multilocus genotype (MLG) was detected in the samples studied.

Clonal reproduction is often considered an evolutionary dead-end (Stebbins, 1957). Lambertini et al. (2010) detected low genetic diversity in three clonal aquatic species, Elodea canadensis, Egeria densa, and Lagarosiphon major. Ren et al. (2005) reported very low genetic differentiation in Eichhornia crassipes (water hyacinth) populations from different localities in China. Ellstrand and Roose (1987) summarized the population genetic structure of 21 clonal plant species and revealed that two species, Taraxacum obliquum, and Gaura triangulata, did not display any apparent intraspecific variability. Pollux et al. (2007) reported that the shifting balance between sexual and asexual reproduction affected the genotypic diversity within populations *Sparganium emersum*. These studies suggest that the plants reproducing asexually can preserve well-adapted genomes in stable environments, which permits the fixation of well-adapted phenotypes over generations (Niklas and Cobb, 2017).

Various species of *Nymphaea* are known to be propagated by both seeds and bulbils (Wiersema, 1988; Ansari and Jeeja, 2009). The prevalent mode of reproduction (asexual vs. sexual) in these plants may likely influence the extent of genetic variability of the species. Our results showed that sexual reproduction in *N. micrantha* is significantly impaired in all the investigated populations. Manually pollinated flowers were aborted at different stages due to low pollen viability (male fitness), and poor ovule receptivity (female fitness). These results were comparable to the flowers that were marked for natural fruit-set (open-pollination). As seen in the present work as well, the species is known to propagate predominantly through leaf propagules (Wiersema, 1988). The extensive clonality accompanied by impaired sexual reproduction could be the major cause for the observed lack of genetic diversity in *N. micrantha.* Moreover, *N. micrantha* is not indigenous to India (Ansari and Jeeja, 2009; POWO, 2021), although, the time and source of the introduction are not known. It is predicted that populations in their introduced range are less variable relative to their source or native range, owing to founder effects and genetic drift (Husband and Barrett, 1991; Amsellem et al., 2000; Hagenblad et al., 2015). The successful expansion of its distribution range in India demonstrates its strong adaptability to the local ecological conditions. The non-existence of genetic variation within and between *N. micrantha* populations, therefore, may be attributed to (1) predominant vegetative propagation, broad tolerance range, and plasticity of genotypes; (2) introduction of the same genotype once or multiple times in the North-eastern parts of India; (3) fixation of well-adapted genomes over generations.

Our results suggest that a single genotype was introduced to India and expanded its distribution range through massive vegetative reproduction. The ability of *N. micrantha* to conquer water bodies in the North-East regions of India as a silent invader could also be attributed to human interventions. The ornamental and religious values of the species aided by human involvement might have played a role in its spread. Several previous studies have also demonstrated the presence of low genetic diversity in invasive and clonally reproducing plant species supporting our results. Xu et al. (2003) reported extremely low genetic diversity in the invasive alligator weed in China. Li et al. (2006) revealed a lack of genetic variation in the clonally reproducing invasive plant Eichhornia crassipes.

## Conclusion

The importance of *Nymphaea* in wetland ecosystems and their increased vulnerability make them a great choice for conservation and management. This will also be useful for the sustainable use of *Nymphaea* species. The existing re-establishment practices, which include restoration of degraded habitats and introduction of individuals into locations, may help recover the declining populations of *Nymphaea* (Guo et al., 2019). However, the successful establishment will require sufficient genetic variability in introduced populations to avoid inbreeding depression and to cope with environmental changes (Forsman and Wennersten, 2016). In this study, we developed a set of novel SSR markers in *Nymphaea*. These SSR markers were used to study the diversity of two widely distributed species, *N. nouchali*, and *N. micrantha*, and had demonstrated contrasting genetic variation patterns in the two species, which was complemented by our results from the reproductive biology of the two species. This contrast in reproductive strategy impacting the existing genetic diversity is an excellent example of varying reasons in nature that can influence the genetic diversity and, hence, the survival of a species. It also underlines the importance of genetic diversity studies in strategizing conservation practices. The information generated in this study is of important consideration for successful wetland restoration programs and genetic resource conservation in *Nymphaea* species, especially those with limited distribution. High genetic variability detected in *N. nouchali* makes it the species of choice for the successful restoration of wetlands. The two gene pools, cluster I and cluster II, which detected more genetic diversity, can be chosen to procure plant material during the restoration programs, although, regional adaptation should also be taken into consideration.

## Data Availability Statement

The datasets presented in this study can be found in online repositories. The names of the repository/repositories and accession number(s) can be found in the article/Supplementary Material. The raw DNA sequencing data of *Nymphaea* micrantha has been deposited at NCBI SRA database under the BioProject accession number PRJNA750447.

## Author Contributions

SG conceived and designed the experiments, analyzed and interpreted the results, and authored and reviewed drafts of the manuscript. SP collected the plant material, designed and performed lab experiments, performed the statistical analysis, analyzed and interpreted the results, and authored the manuscript. NS collected the plant material and performed lab experiments. AA performed pollen biology and ovule receptivity tests and wrote the manuscript. SK designed the experiments and organized the collection of plant material. RT designed the experiments, analyzed and interpreted the results, and wrote the manuscript. MA and AJ participated in designing lab experiments and reviewed the drafts of the manuscript. All authors have read and approved the manuscript.

## Conflict of Interest

The authors declare that the research was conducted in the absence of any commercial or financial relationships that could be construed as a potential conflict of interest.

## Publisher’s Note

All claims expressed in this article are solely those of the authors and do not necessarily represent those of their affiliated organizations, or those of the publisher, the editors and the reviewers. Any product that may be evaluated in this article, or claim that may be made by its manufacturer, is not guaranteed or endorsed by the publisher.

## Footnotes

1
https://www.bioinformatics.nl/cgi-bin/primer3plus/primer3plus.cgi

## Supplementary Material

Supplementary Figure 1 Heat map analysis plot of Fisher’s exact test (goodness-of-fit) for Hardy-Weinberg (HW) equilibrium generated using the hw.test function from the pegas package in R for each locus in each population. All loci in pink are loci suspected to be deviated from HWE with *p* ≤ 0.05.

Supplementary Figure 2 Histogram showing relative DNA content of *Nymphaea micrantha* (peak A, coefficient of variation 4.92 %) and the reference, *Solanum lycopersicum* L. “Stupicke’polnı’ rane”’ (reference, peak B, coefficient of variation 6.02 %) obtained after analysis of nuclei isolated from young leaves using CyFlow^®^ Cube 8 flow cytometer (Sysmex, Germany). % CV, coefficient of variation of the peaks as percentage (Standard deviation of the peak/mean position of the peak × 100).

Supplementary Figure 3 Assembly statistics of *Nymphaea micrantha* genome before and after length filtration.

Supplementary Figure 4 Spatial autocorrelation correlogram for total population (Omega = 157.275, *p* = 0.00) and four genetic clusters inferred from DAPC. Solid line indicates autocorrelation (−1 < *r* < +1) between individuals at each distance; dashed lines indicate 95 % confidence region around the null hypothesis of no genetic structure (*r* = 0) based on 10,000 permutations. Error bars for each distance class resulted from 10,000 bootstraps of individuals. Values outside the upper (U) and lower (L) 95% confidence interval indicate significant spatial structure.

Supplementary Figure 5 Decline in the population of *Nymphaea* species in different parts of Goa, Maharashtra, and Kerala due to construction (top left), habitat destruction by the local people (top right), excessive growth of wild grasses (bottom left) and water pollution (bottom right).

Supplementary Table 1 Details of 260 SSR primers (217 non-genic + 43 genic) designed in present study.

Supplementary Table 2 Observations made using an epifluorescence microscope to test pollen fertility and viability in *Nymphaea micrantha*.

Supplementary Table 3 Assembly statistics of *Nymphaea micrantha* genome.

Supplementary Table 4 Contigs containing simple sequence repeats in *Nymphaea* genome.

Supplementary Table 5 Annotation details of 12556 non-redundant SSR containing sequences.

Supplementary Table 6 Cross species amplification of 57 polymorphic SSR loci in *Nymphaea*.

Supplementary Notes Supplementary notes including supplementary findings of microsatellites discovery in *Nymphaea micrantha* genome and cross-species transferability of polymorphic SSR markers discovered from the study.
