# Supplementary figures and images for: Contrasting Reproductive Strategies of Two Nymphaea Species Affect Existing Natural Genetic Diversity as Assessed by Microsatellite Markers: Implications for Conservation and Wetlands Restoration
Source: Front Plant Sci. 2022 Mar 9;13:773572. doi: 10.3389/fpls.2022.773572 (PMC8965595; doi:10.3389/fpls.2022.773572)

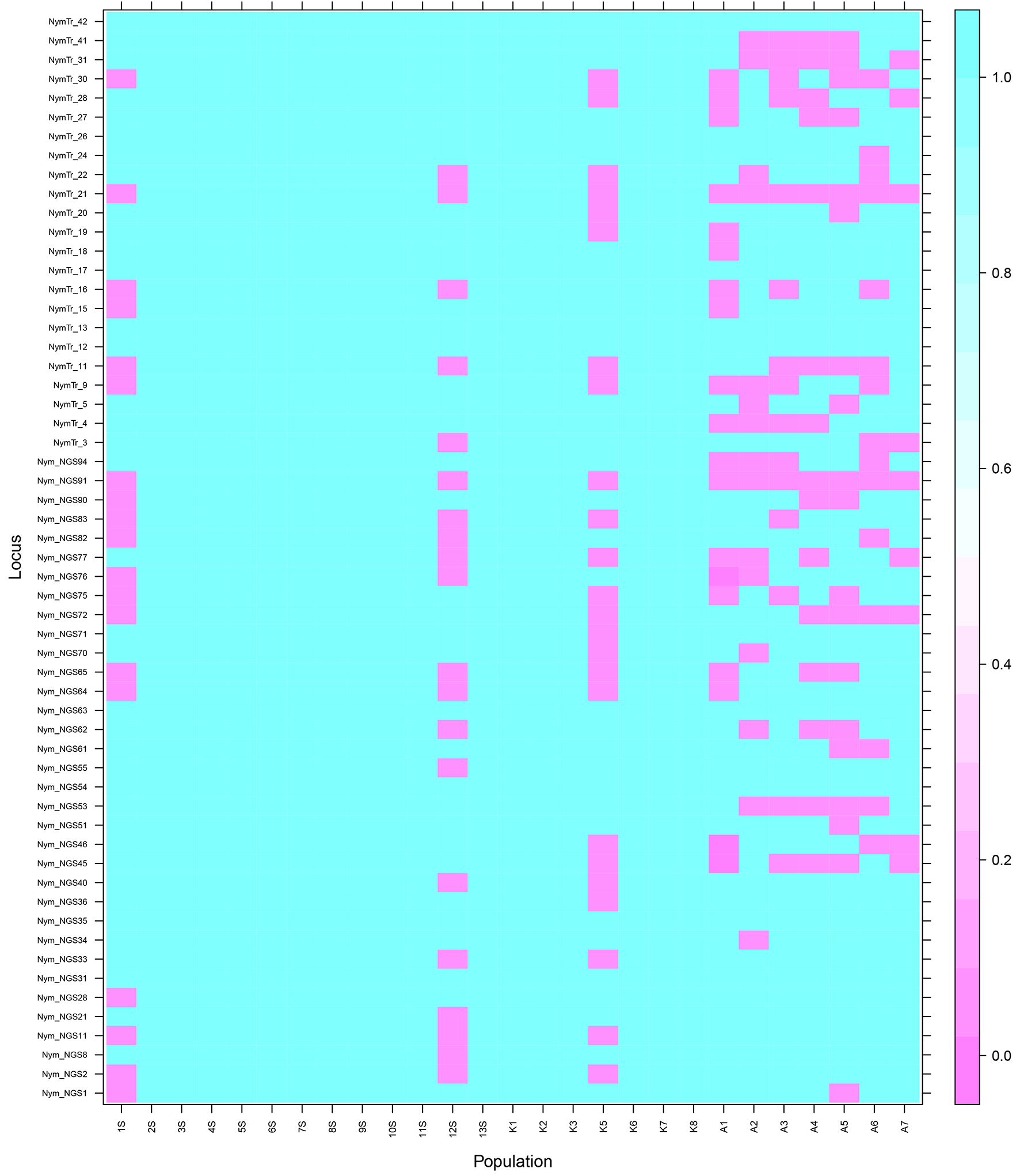

Supplement: Supplementary Figure 1 — Heat map analysis plot of Fisher’s exact test (goodness-of-fit) for Hardy-Weinberg (HW) equilibrium generated using the hw.test function from the pegas package in R for each locus in each population. All loci in pink are loci suspected to be deviated from HWE with p ≤ 0.05. [file Image_1.TIF]

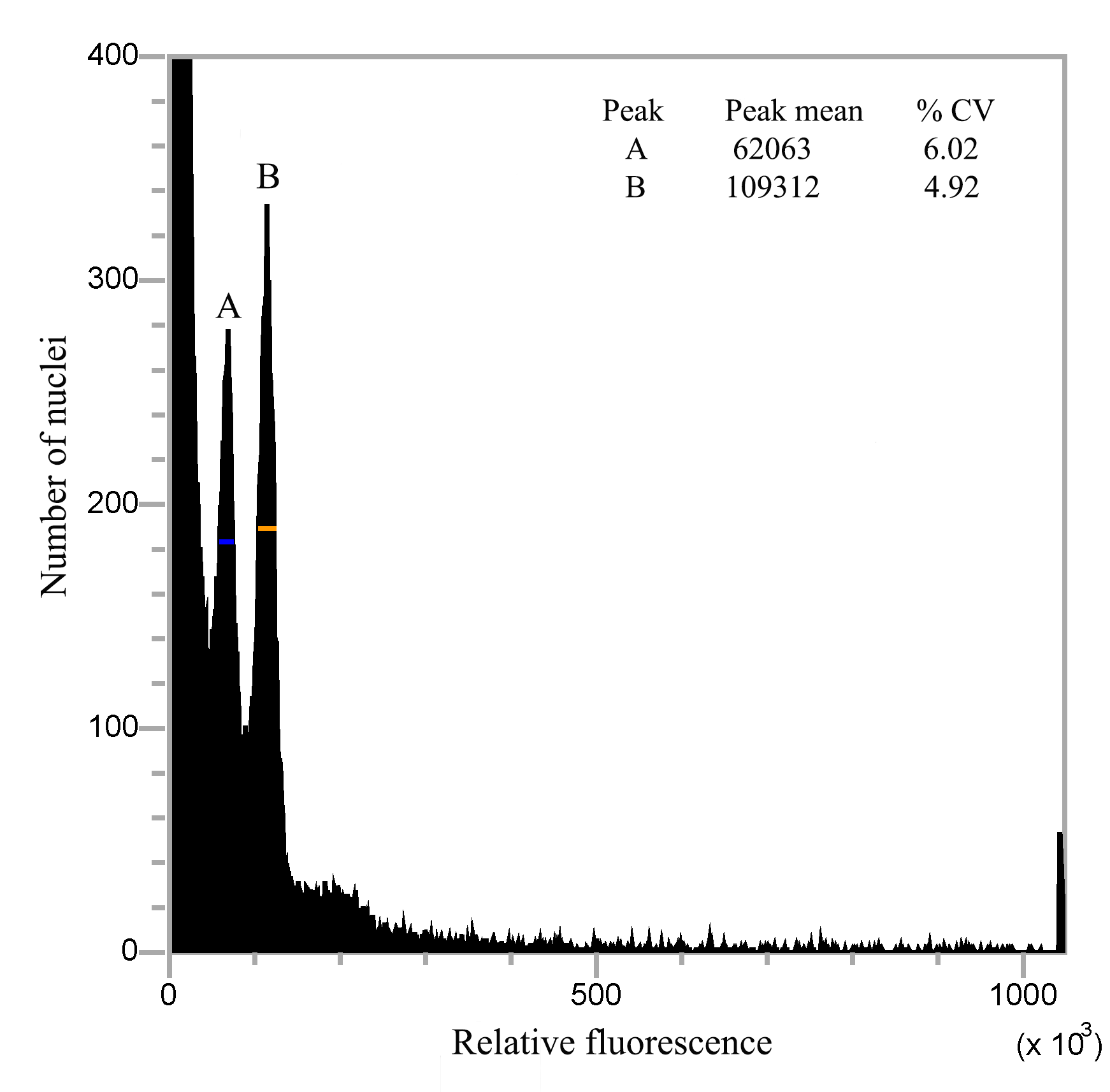

Supplement: Supplementary Figure 2 — Histogram showing relative DNA content of Nymphaea micrantha (peak A, coefficient of variation 4.92 %) and the reference, Solanum lycopersicum L. “Stupicke’polnı’ rane”’ (reference, peak B, coefficient of variation 6.02 %) obtained after analysis of nuclei isolated from young leaves using CyFlow® Cube 8 flow cytometer (Sysmex, Germany). % CV, coefficient of variation of the peaks as percentage (Standard deviation of the peak/mean position of the peak × 100). [file Image_2.TIF]

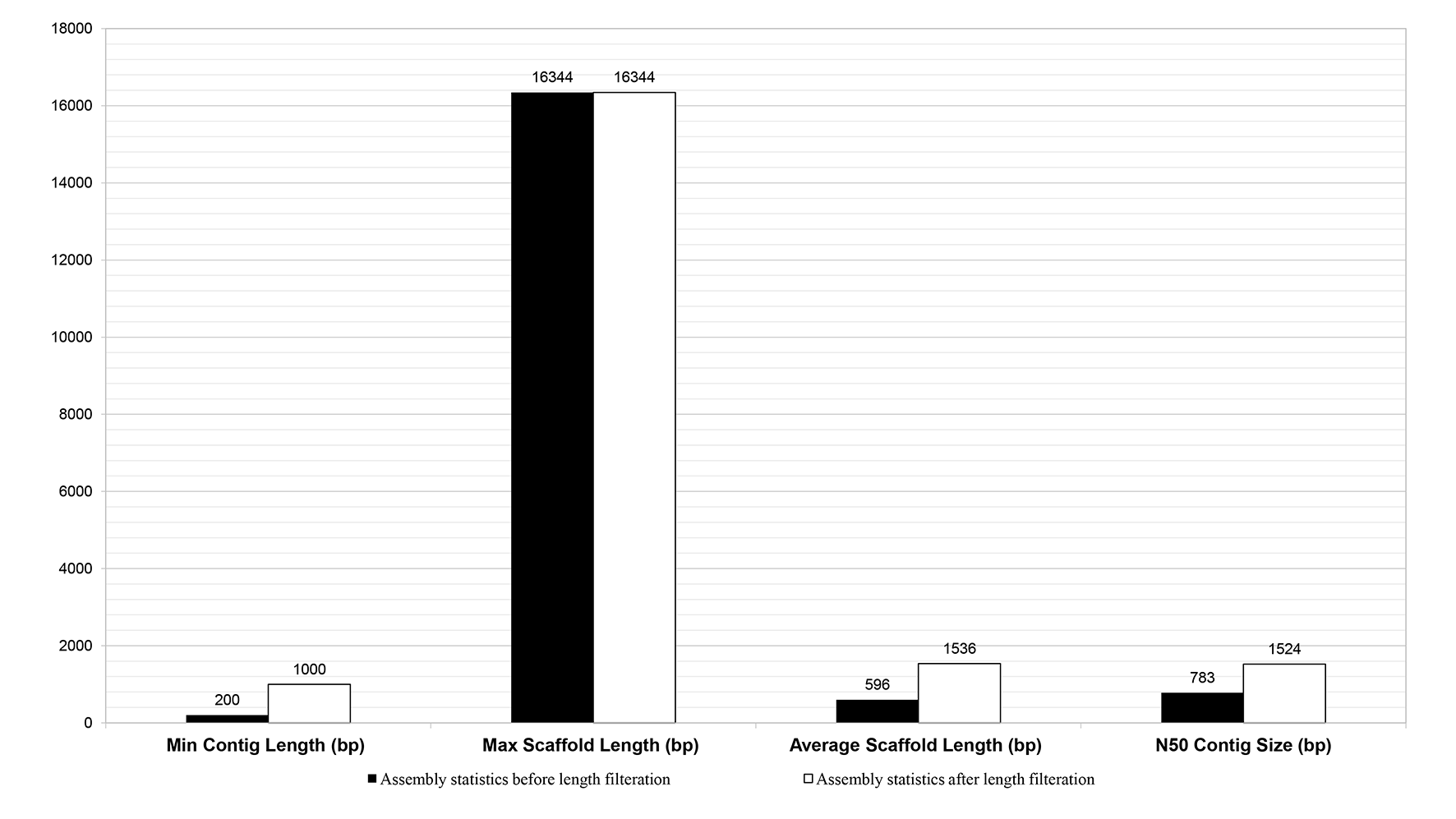

Supplement: Supplementary Figure 3 — Assembly statistics of Nymphaea micrantha genome before and after length filtration. [file Image_3.TIF]

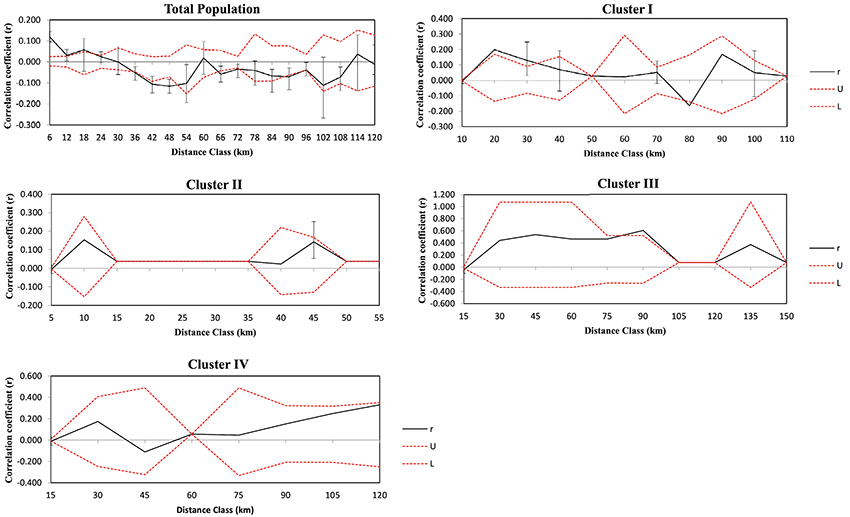

Supplement: Supplementary Figure 4 — Spatial autocorrelation correlogram for total population (Omega = 157.275, p = 0.00) and four genetic clusters inferred from DAPC. Solid line indicates autocorrelation (−1 < r < +1) between individuals at each distance; dashed lines indicate 95 % confidence region around the null hypothesis of no genetic structure (r = 0) based on 10,000 permutations. Error bars for each distance class resulted from 10,000 bootstraps of individuals. Values outside the upper (U) and lower (L) 95% confidence interval indicate significant spatial structure. [file Image_4.TIFF]

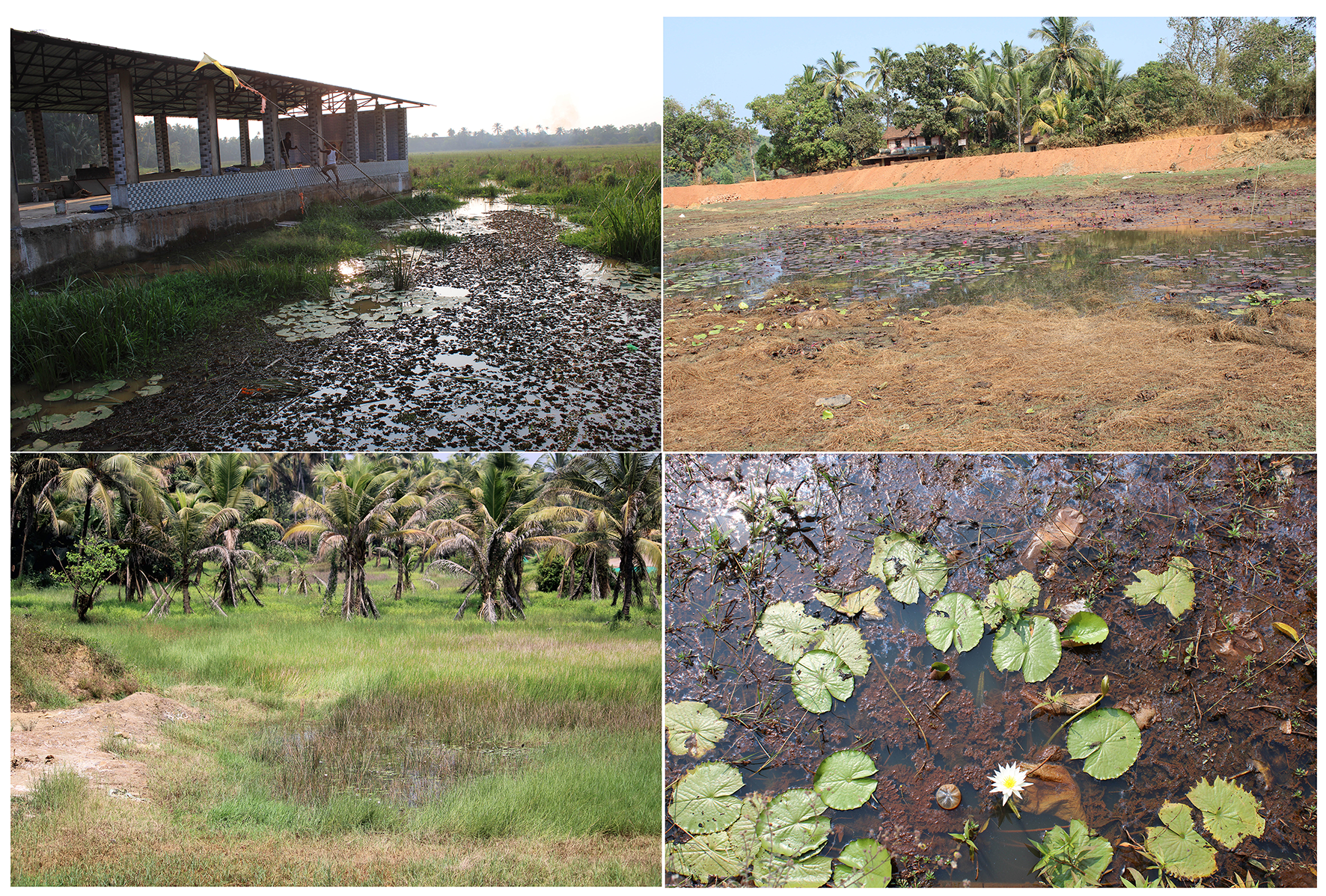

Supplement: Supplementary Figure 5 — Decline in the population of Nymphaea species in different parts of Goa, Maharashtra, and Kerala due to construction (top left), habitat destruction by the local people (top right), excessive growth of wild grasses (bottom left) and water pollution (bottom right). [file Image_5.TIF]
