# Supplementary material for: Contrasting Reproductive Strategies of Two Nymphaea Species Affect Existing Natural Genetic Diversity as Assessed by Microsatellite Markers: Implications for Conservation and Wetlands Restoration
Source: Front Plant Sci. 2022 Mar 9;13:773572. doi: 10.3389/fpls.2022.773572 (PMC8965595; doi:10.3389/fpls.2022.773572)
Supplement: Supplementary Notes — including supplementary findings of microsatellites discovery in Nymphaea micrantha genome and cross-species transferability of polymorphic SSR markers discovered from the study. [file Data_Sheet_1.DOCX]

1. **Genome sequencing and discovery of microsatellites**

Genome sequencing and assembly resulted 77175 contigs with an average contig length of 1536bp (Supplementary Table 4). The assembled contigs were used to discover the distribution of simple sequence repeats (SSRs) using the Perl script misa.pl and characterized for different repeat types. SSRs with di-nucleotide repeats were the most frequent type representing 74.85% of total SSRs, followed by tri- (11.89%), mono- (5.97%) tetra- (5.13%), penta- (1.3%), and hexa-nucleotide repeats (0.84%) (Figure S1). In *Nymphaea* genome, the most abundant repeat type was AG/CT accounting for 47.08%, followed by AT/TA (38.45%) and AC/GT (14.44%) repeats. Only four CG/GC repeat loci were obtained in the analyzed data. Among trinucleotide repeats, AAG/TTC was the most frequent motif (42%) followed by AAT/TTA (23%) and AGG/TCC (8.5%). Among tetranucleotide repeats, AAAT/TTTA repeat motif (36 %) was the most frequent type followed by AAAG/TTTC (14.43 %) and ACAT/TGTA (12.96 %). Among penta- and hexa-nucleotide repeats, AAAAT/AAAGG (15.22 %) and AAAGAG (13.16 %) respectively were the most frequent motif types. The reiteration number of the SSR motifs also varied and ranged from 4 to 26. Maximum number of reiteration units was for dinucleotide motifs followed by tri-, tetra-, penta- and hexa-nucleotide motifs.

| **Identification of simple sequence repeats (SSRs) in *Nymphaea micrantha* genome.** | |
| --- | --- |
| Total number of sequences examined | 77175 |
| Total size of examined sequences (bp) | 118553353 |
| Total number of identified SSRs | 22268 |
| Number of SSR containing sequences | 16937 |
| Number of sequences containing more than 1 SSR | 4123 |
| Number of SSRs present in compound formation | 2464 |

**
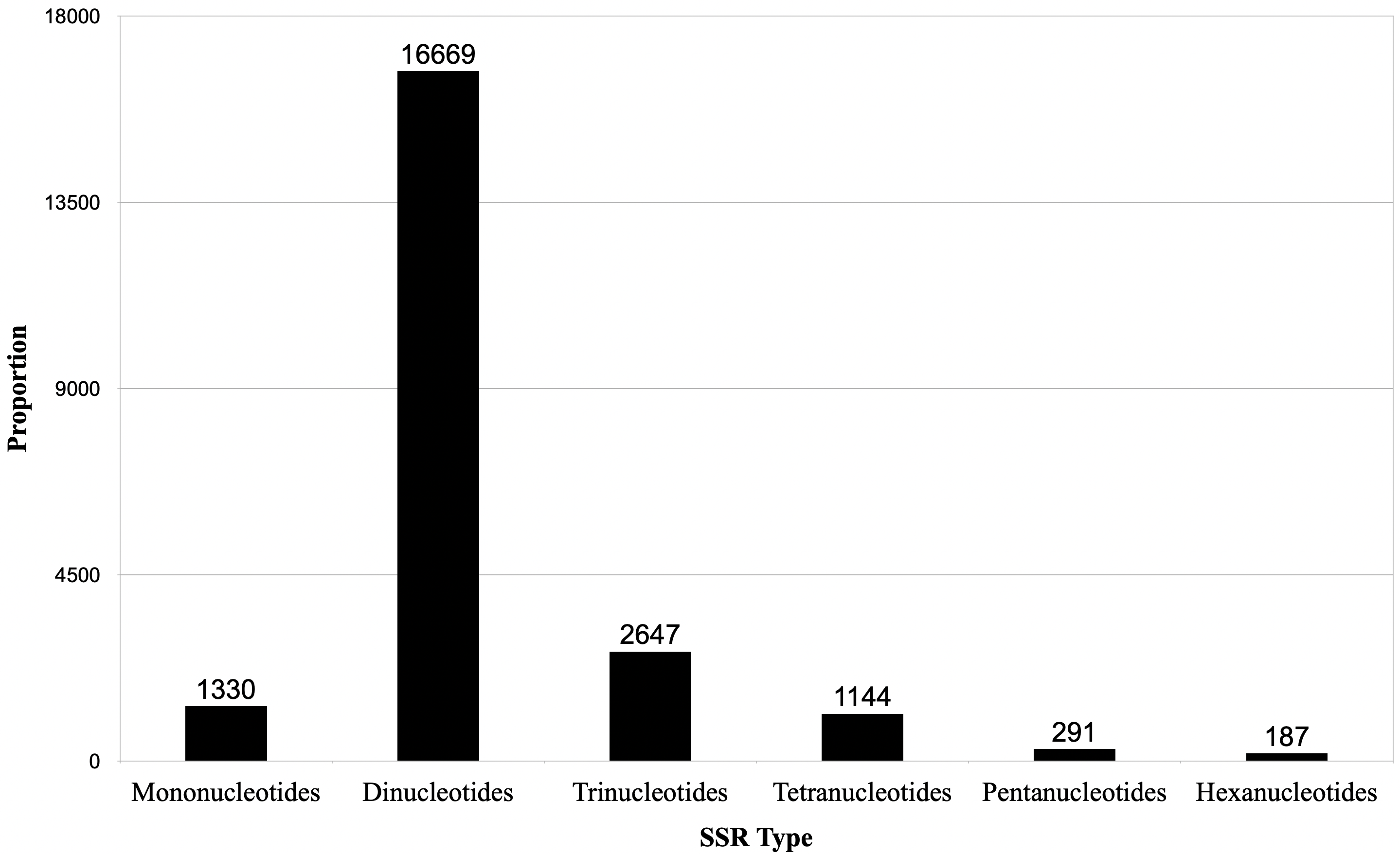
**

**Figure S1:** Identification of simple sequence repeats (SSRs) and the frequency distribution of different classes of SSRs in the *Nymphaea* genome.

1. **Cross-species transferability and phylogenetic relationships between *Nymphaea* species**

*2.1 Cross-species amplification of polymorphic SSR markers*

Amplification of the 57 polymorphic microsatellite markers studied in *N. micrantha* and *N. nouchali* was also assessed in five other *Nymphaea* species (Supplementary Table 2). Each primer pair, except Nym_NGS46, was found to be amplifiable in one or more of the tested *Nymphaea* species. Ten SSR loci (Nym_NGS1, Nym_NGS7, Nym_NGS11, Nym_NGS55, Nym_NGS62, Nym_NGS76, NymTr_11, NymTr_24, NymTr_26, NymTr_28) were able to produce bands in all the species showing 100 % transferability. The highest cross-species transferability rate was observed in *N. malabarica* (96.49 %) followed by *N. caerulea* (91.23 %), *N. rubra* (56.14 %), *N. pubescens* (49.12 %) and *N. × khooroi* (38.60 %) (see Supplementary Table 2).

*2.2 Phylogenetic network analysis*

Phylogenetic networks provide an alternative to phylogenetic trees and are more suitable to represent evolutionary trajectories for datasets whose evolution involves reticulate events such as hybridization, horizontal gene transfer, or recombination (Huson & Bryant, 2006). Various research groups have unraveled reticulate evolutionary patterns in *Nymphaea* spp. (Borsch et al., 2007; Löhne et al., 2008). Therefore, because the datasets for which reticulate events are detected are better represented by networks than tress, the cross-species transferable polymorphic SSR markers were used to compute phylogenetic network among the studied species. Analysis was performed with SplitsTree5 (Huson, 1998, Huson and Bryant 2006) using Neighbor-Net method (Bryant & Moulton, 2004) to obtain splits and Splits Network Algorithm method (Dress & Huson, 2004) to obtain the splits network.


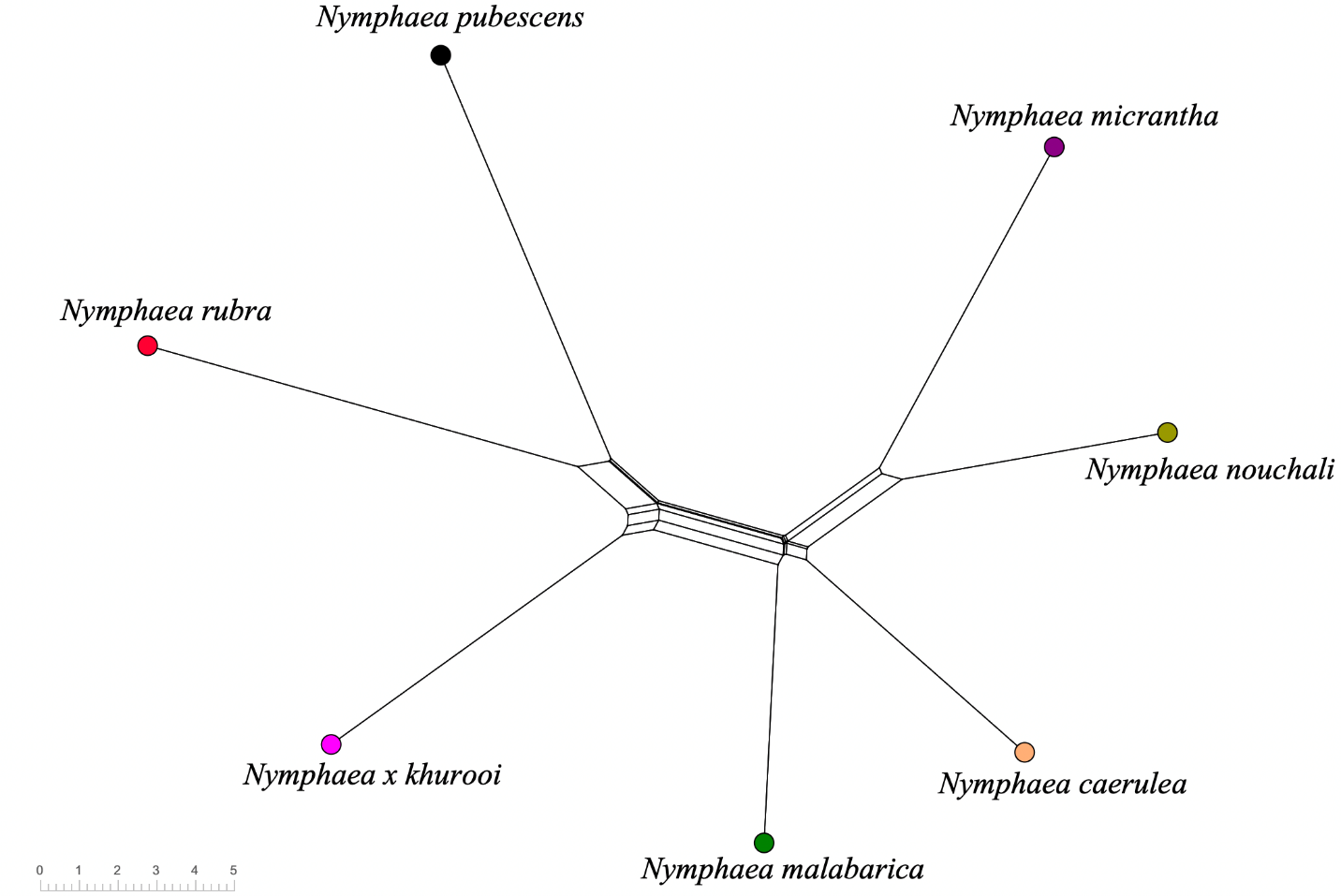


**Figure S2:** Phylogenetic network of the *Nymphaea* species constructed with SplitsTree5 (Huson and Bryant, 2006) using the cross-species transferable microsatellite markers. Clustering is based on Neighbor-net algorithm (Bryant and Moulton, 2004). Long parallel edges represent consistent phylogenetic information.

The Neighbor-net splits network (Figure S2) supported the split of species belonging to the same subgenus into one group. *N. micrantha*, *N. nouchali* and *N. caerulea*, which belong to the subgenus *Brachyceras* were more closely related. *N. rubra* and *N. pubescens* belonging to the subgenus *Lotus* were found to be more closely related to *N. × khurooi* subgenus *Nymphaea*.

**References**

Borsch, T., Hilu, K. W., Wiersema, J. H., Löhne, C., Barthlott, W., & Wilde, V. (2007). Phylogeny of Nymphaea (Nymphaeaceae): evidence from substitutions and microstructural changes in the chloroplast trnT-trnF region. *International Journal of Plant Sciences*, *168*(5), 639-671.

Bryant, D., & Moulton, V. (2004). Neighbor-net: an agglomerative method for the construction of phylogenetic networks. *Molecular biology and evolution*, *21*(2), 255-265.

Dress, A. W., & Huson, D. H. (2004). Constructing splits graphs. *IEEE/ACM transactions on Computational Biology and Bioinformatics*, *1*(3), 109-115.

Huson, D. H. (1998). SplitsTree: analyzing and visualizing evolutionary data. *Bioinformatics (Oxford, England)*, *14*(1), 68-73.

Huson, D. H., & Bryant, D. (2006). Application of phylogenetic networks in evolutionary studies. *Molecular biology and evolution*, *23*(2), 254-267.

Löhne, C., Borsch, T., Jacobs, S. W., Hellquist, C. B., & Wiersema, J. H. (2008). Nuclear and plastid DNA sequences reveal complex reticulate patterns in Australian water-lilies (Nymphaea subgenus Anecphya, Nymphaeaceae). *Australian Systematic Botany*, *21*(4), 229-250.
